# Rhodanine derived enethiols react to give 1,3-dithiolanes and mixed disulfides†

**DOI:** 10.1039/d4md00157e

**Published:** 2024-06-05

**Authors:** Jos J. A. G. Kamps, Dong Zhang, Timothy D. W. Claridge, Christopher J. Schofield

**Affiliations:** a Chemistry Research Laboratory, Department of Chemistry and the Ineos Oxford Institute for Antimicrobial Research, University of Oxford Oxford OX1 3TA UK christopher.schofield@chem.ox.ac.uk

## Abstract

Rhodanines have been characterised as ‘difficult to progress’ compounds for medicinal use, though one rhodanine is used for diabetes mellitus treatment and others are in clinical development. Rhodanines can undergo hydrolysis to enethiols which are inhibitors of metallo-enzymes, such as metallo β-lactamases. We report that in DMSO, rhodanine derived enethiols undergo dimerisations to give 1,3-dithiolanes and mixed disulfides. The results highlight the potential of rhodanines and enethiols to give multiple products. They suggest that where possible DMSO should be avoided as a storage solvent for rhodanines/enethiols and highlight the need for further research on biologically relevant enethiols/mixed disulfides.

## Abstract 2

Unexpected reaction of enethiols to form 1,3-dithiolanes and mixed disulfides in DMSO occur through a common thioketone intermediate.

## Graphical abstract

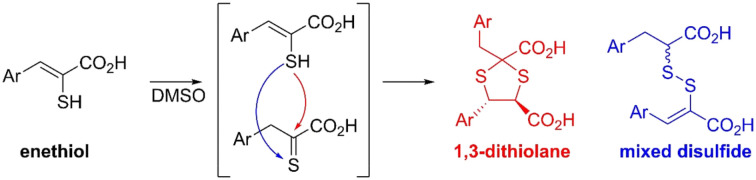

## Introduction

**Fig. 1:**
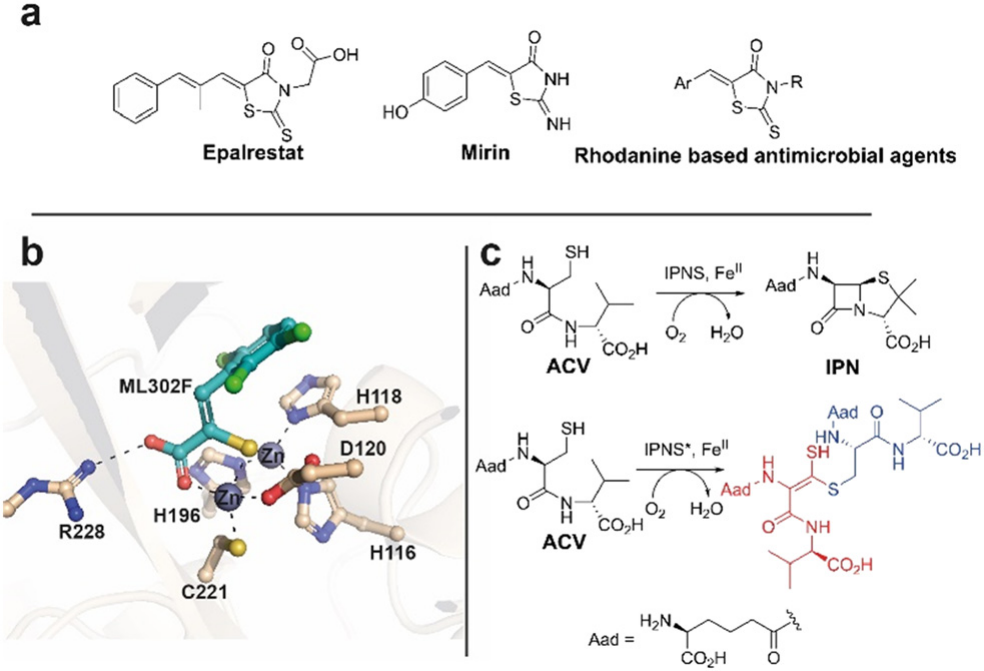
Examples of rhodanines and enethiols in medicinally related chemistry. (a) Epalrestat, mirin, and rhodanine based antimicrobial agents. (b) Hydrolysis of rhodanines occurs in a pH dependent manner to form enethiols which inhibit MBLs by binding to their active site zinc ions, as shown for ML302F binding to the VIM-2 MBL.^14,15^ (c) Isopenicillin-N synthase (IPNS) catalysed conversion of l-δ-(α-aminoadipoyl)-l-cysteinyl-d-valine (ACV) into isopenicillin N (IPN). Incubation of ACV with a C-terminally truncated variant of IPNS results in the formation of an enethiol disulfide product.^16^ Aad: l-δ-(α-aminoadipoyl).

**Fig. 2:**
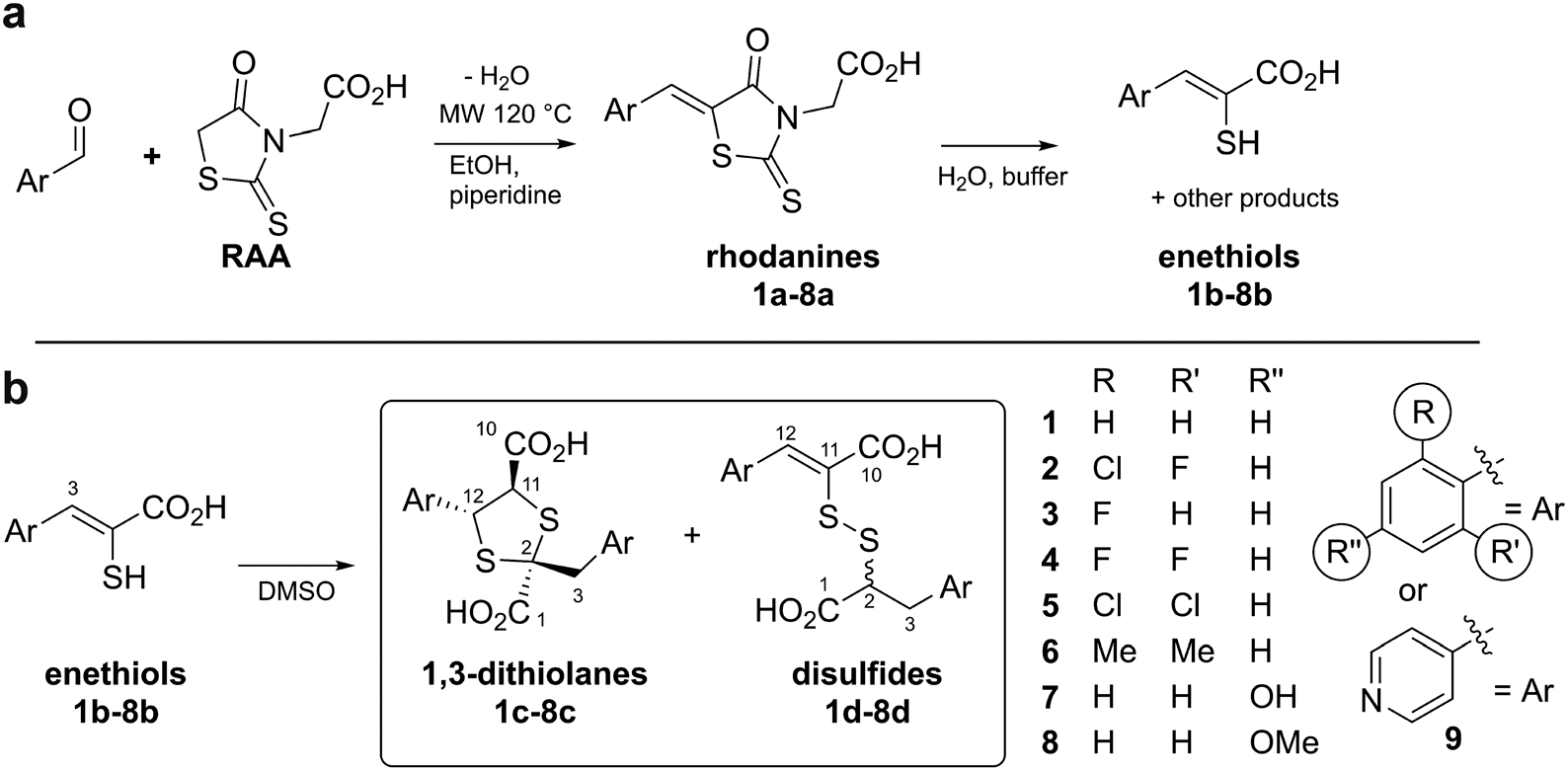
Preparation and reaction of rhodanines and enethiols. (a) Preparation of rhodanines from the corresponding aldehyde and rhodanine acetic acid (RAA), and their hydrolysis to give enethiols.^14,15^ (b) Enethiols react in DMSO to form 1,3-dithiolanes and mixed disulfides as described in this work. Note all compounds are racemic and all stereochemical assignments are provisional (see text).

Rhodanines feature in many medicinal chemistry studies, but have also been characterised both as ‘privileged’ scaffolds and as ‘promiscuous’ inhibitors.^1,2^ One rhodanine, epalrestat, an aldose reductase inhibitor, is used for diabetes mellitus treatment (Fig. 1a).^3–5^ Mirin^6^ and rhodanine derivatives are also used in DNA damage repair research.^7,8^ Rhodanines are reported to manifest antimicrobial activity by inhibition of penicillin binding proteins^9,10^ and inhibit serine β-lactamases^11^ and metallo β-lactamases (MBLs).^12,13^ In work on metallo β-lactamase (MBL) inhibition,^14,15^ we found that activity originally ascribed to rhodanines,^11,13^ is, at least in part, due to hydrolysis of rhodanines to give enethiols (Fig. 2a), which chelate the MBL active site Zn(ii) ions (Fig. 1b). In some cases, we accrued crystallographic evidence that enethiols and rhodanines can simultaneously bind at an MBL active site. Rhodanine hydrolysis occurs spontaneously in water and is pH dependent (Fig. 2a).^15^ Enethiol derivatives have also been identified as enzyme products and intermediates,^16–19^ for example as formed on incubating l-δ-(α-aminoadipoyl)-l-cysteinyl-d-valine (ACV) and substrate analogues with a C-terminally truncated isopenicillin N synthase variant (Fig. 1c).^16^

During studies on rhodanine derived enethiol solutions in DMSO-d_6_,^14,15^ we observed that freshly acquired ^1^H NMR spectra manifested the anticipated enethiol resonances, but on storage in DMSO-d_6_ further time dependent reactions occur.

Given the biomedicinal interest in rhodanines and enethiols, we carried out studies to investigate the identities of the enethiol derived products. The results reveal enethiols dimerise to give 1,3-dithiolanes and mixed disulfides as major products, with the observed ratio depending on the nature of the enethiol substituents (Fig. 2b).

## Results

The enethiol starting materials (1b–8b) were prepared as reported, wherein Knoevenagel condensation of an aldehyde and rhodanine-3-acetic acid (RAA), is followed by basic hydrolysis.^14,15^

**Fig. 3:**
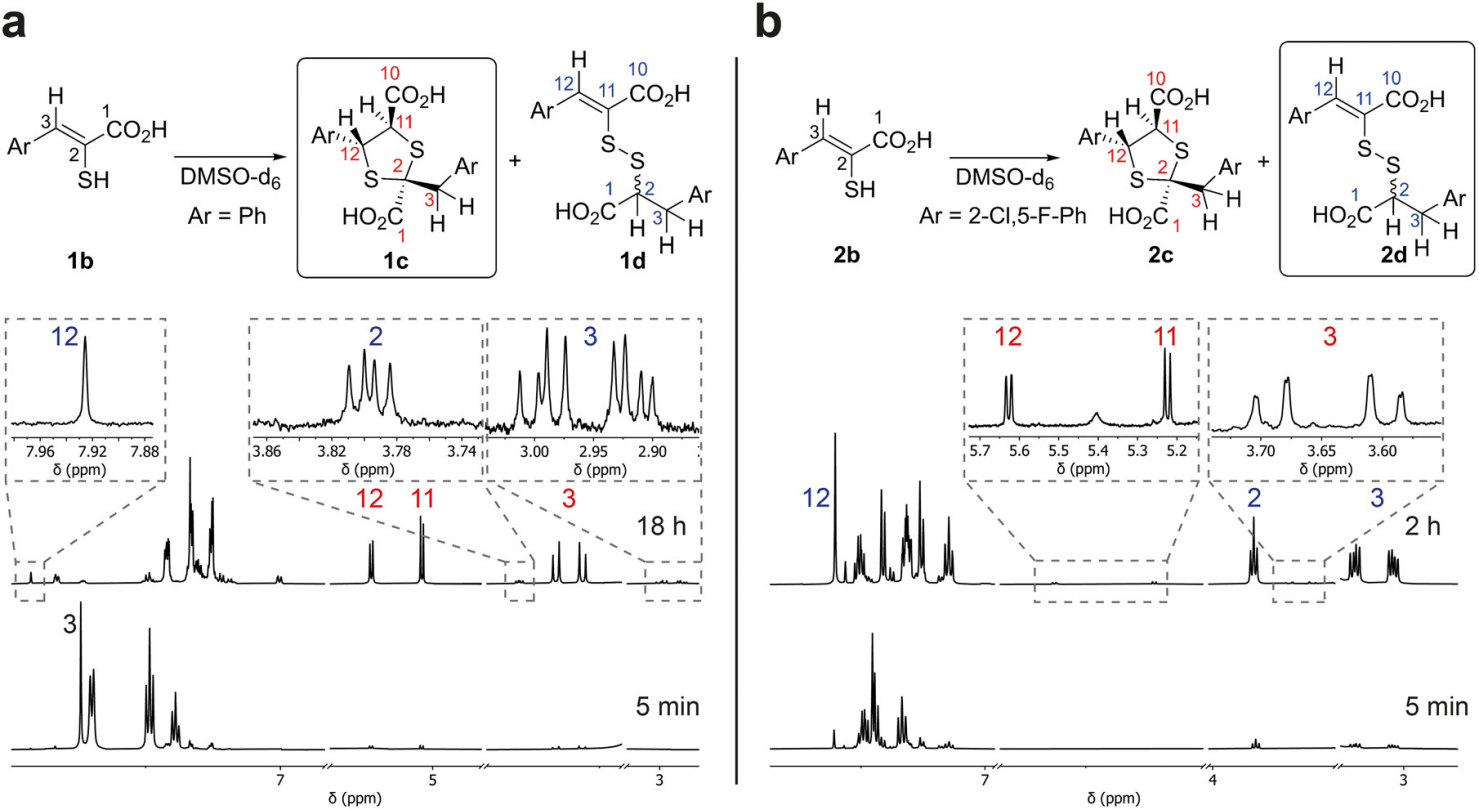
Reaction of enethiols 1b (a) and 2b (b) in DMSO-d_6_ as analysed by ^1^H NMR (700 MHz). Boxed structures represent the major observed products. Selected resonances are labelled with numbers to match the protons in the structures, with red and blue numbers representing the 1,3-dithiolanes and the mixed disulfides, respectively. Note all compounds are racemic and stereochemical assignments are provisional (see text).

Monitoring of the reaction of enethiol 1b in DMSO-d_6_ over time by ^1^H NMR (700 MHz) led to assignment of 1,3-dithiolane 1c as the major observed product (Fig. 3). Thus, a reduction in the intensity of the 1b H-3 singlet (*δ*_H_ 7.74 ppm) correlated with formation of two doublets corresponding to 1c H-12 and 1c H-11 (*δ*_H_ 5.23 and 5.04 ppm (*J* = 6.5 Hz), respectively) and two doublets corresponding to 1c H_2_-3 (*δ*_H_ 3.66 and 3.56 ppm (*J* = 14.0 Hz)). LC-MS analyses of the NMR solution demonstrated a mass of 361 *m*/*z* ([M + 1]^+^) consistent with formation of 1c. ^1^H–^13^C-heteronuclear single quantum correlation NMR (HSQC) and ^1^H–^13^C-heteronuclear multiple bond correlation NMR (HMBC) analyses revealed that the 1c H_2_-3 doublets (*δ*_H_ 3.66 and 3.56 ppm, *δ*_C_ 43.8 ppm) and the 1c H-12 and 1c H-11 doublets (*δ*_H_ 5.23 and 5.04 ppm, *δ*_C_ 58.2 and 63.0 ppm respectively) manifest through bond couplings to quaternary carbon 1c S–C̲–S-2 (*δ*_C_ 67.4 ppm), supporting 1,3-dithiolane 1c formation (Fig. 3 and S1†). The stereochemistry of the 1,3-dithiolane was not assigned based on our NMR studies, though it is likely one major product is present; the 1,3-dithiolane stereochemistry shown in figures is based on prior precedent including crystallographic studies as reported by Castiñeiras *et al.*^20^

By contrast, NMR analyses of reaction of enethiol 2b in DMSO-d_6_, revealed the formation of a different type of major observed product, which was assigned as the mixed disulfide 2d; with lower levels of 1,3-dithiolane 2c being observed (Fig. 3 and S2†). Further investigation of the reaction of 1b indicated that in addition to 1c, mixed disulfide 1d is formed albeit at a lower (∼6%) observed level than for formation of 2d from 2b (the low levels of 1d and 2c, precluded their full assignment by HSQC and HMBC NMR).

After reaction of 2b for 2 hours, the ^1^H NMR spectrum manifested a singlet corresponding to 2d H-12 (*δ*_H_ 7.60 ppm) formed together with an ABX system; an apparent triplet corresponding to 2d H-2 (*δ*_H_ 3.83 ppm (*J* = 8.0 Hz)) and two double doublets corresponding to 2d H_2_-3 (*δ*_H_ 3.20 and 3.04 ppm (*J* = 14.0 and 8.0 Hz)) (Fig. 3b).

^19^F NMR (565 MHz; proton-coupled) time course studies showed the disappearance of a broad singlet (*δ*_F_ −107.3 ppm) corresponding to 2b and appearance of two apparent triplets (*δ*_F_ −107.5 and −111.5 ppm (*J* = 7.5 Hz)) supporting the unexpected formation of the mixed disulfide 2d (Fig. S3†). LC-MS analyses manifested a mass of 465 *m*/*z* ([M + H]^+^), also consistent with formation of mixed disulfide 2d. HSQC and HMBC analyses of the reaction mixture demonstrated that the 2d H_2_-3 doublets (*δ*_H_ 3.20 and 3.04 ppm, *δ*_C_ 29.4 ppm) couples with 2d carbons 1, 2, 4, 5, and 9, and the 2d H-2 triplet (*δ*_H_ 3.83 ppm, *δ*_C_ 53.3 ppm) couples with 2d carbons 1, 3, and 4 (Fig. S2†). The HSQC and HMBC NMR demonstrated through-bond connectivity between 2d H-12 (*δ*_H_ 7.60 ppm, *δ*_C_ 134.3 ppm) to 2d carbons 10, 13, 14, and 18. No through-bond coupling between 2d H-2 and C-11 was observed by HMBC analysis, consistent with mixed disulfide formation. Note the stereochemistry of the double bond in the mixed disulfides was not assigned based on our NMR studies.

Formation of 1c/1d and 2c/2d from 1b and 2b, respectively, was also observed under (near) anaerobic (<2 ppm) conditions, implying O_2_ is not involved in the reactions (Fig. S4 and S5†). Enethiol 1b was more stable in tested solvents other than DMSO-d_6_, *i.e.* methanol-d_4_, THF-d_8_, acetone-d_6_, DMF-d_7_, and 1,4-dioxane-d_8_ (Fig. S6–S10†), although partial conversion of 1b to 1c was observed in DMF-d_7_ (∼50% after 72 h, Fig. S9†) (full conversion of 1b in DMSO-d_6_ was observed within 24 h). In some cases, on prolonged incubation we observed ^1^H NMR evidence for potential low-level formation of symmetrical disulfides, possibly arising from the initially formed mixed disulfides. Nonetheless, in all cases where reaction to give disulfides occurred, the mixed disulfides were the major disulfides observed.

**Fig. 4:**
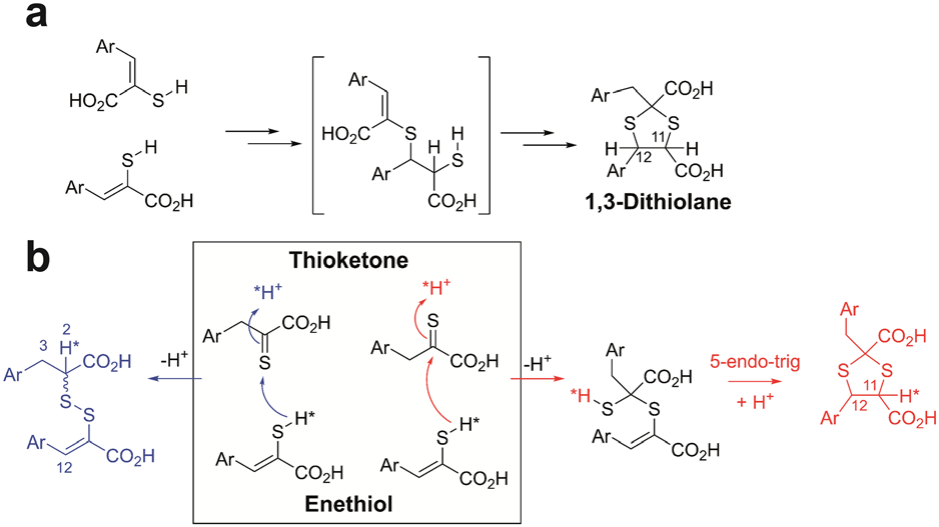
Possible mechanisms for formation of 1,3-thiolanes and mixed disulfides from enethiols. (a) It was previously proposed that sequential electrophilic additions occur (note the proposed intermediate has not been observed).^20^ (b) Alternatively, an enethiol S may react with the C of a thioketone with subsequent 5-*endo-trig* cyclisation giving a 1,3-dithiolane (red pathway). The enethiol S may also react with the S of a thioketone leading to a mixed disulfide (blue pathway). *Indicates predicted preferred positions for incorporation of ^2^H (*H) into the products from incubation with ^2^H_2_O. We observed preferential incorporation of ^2^H at the H-11 position of the 1,3-dithiolane and the H-2 position of the mixed disulfide, consistent with mechanism (b) being preferred. Mechanism (a) would imply similarly high incorporation of ^2^H at both the H-11 and H-12 positions, which we did not observe. Stereochemical preferences are unknown.

Performing the reaction of enethiol 1b at higher concentrations (*i.e.* 10, 50, and 100 mM) in DMSO, increased the rate of formation of 1c and 1d (Fig. S11–S14†). Reaction of 1b to 1c in DMSO-d_6_ with ^2^H_2_O (20 μL ^2^H_2_O in 450 μL DMSO-d_6_) provided evidence for 1,3-dithiolane 1c formation with high (>95%) incorporation of ^2^H at H-11 (as evidenced by loss of the 1c H-11 doublet (*δ*_H_ 5.02 ppm), and the observation of a 1c H-12 singlet (*δ*_H_ 5.21 ppm) (Fig. S15†), with lower incorporation at 1c H-12 and H-3 Fig. S16†). The reaction of 2b in DMSO-d_6_ with ^2^H_2_O (20 μL ^2^H_2_O in 450 μL DMSO-d_6_) provided evidence for formation of mixed disulfide 2d with high (>95%) incorporation of ^2^H at the H-2 position of the reduced component (>95%, Fig. S17†) with lower levels at the H-3 and H-12 positions (Fig. S18†). These observations have mechanistic implications as described in the conclusions (Fig. 4).

To investigate the influence of the substitutions on enethiol reactions, the 2-fluorophenyl 3b, 2,6-difluorophenyl 4b, 2,6-dichlorophenyl 5b, 2,6-dimethylphenyl 6b, 4-hydroxyphenyl 7b, and 4-methoxyphenyl 8b derivatives were investigated (Fig. S19–S24 and S25–S32†). Although more detailed kinetic studies are required, the results show 3b, 4b, and 5b reacted relatively efficiently, giving the corresponding 1,3-dithiolanes (3c, 4c, 5c) and/or mixed disulfides (3d, 4d, 5d; Fig. S27–S29†). Both 7b (4-hydroxyphenyl) and 8b (4-methoxyphenyl) were apparently less reactive (Fig. S31 and S32†) and 2,6-dimethylphenyl 6b was largely unreacted after 24 hours, with only low-level formation of 6d (Fig. S30†).

## Conclusions

The results show rhodanine derived C-3 aryl substituted enethiols react to give both 1,3-dithiolanes and mixed disulfides as major products in DMSO. Both the reaction rate and ratio of 1,3-dithiolane to mixed disulfide products formed are dependent on the aryl substitution pattern. Of the solvents tested the reaction was particularly efficient in DMSO, though more limited reaction was also observed in DMF.

The combined observations show that the reaction outcomes of rhodanines and rhodanine derived enethiols are condition and substitution pattern dependent. They do not preclude the use of such compounds for biological applications. They do, however, reinforce the need to take care in assigning the active component(s) derived from rhodamine precursors, in studies both with isolated targets and in cells/*in vivo*. It should be also noted that the biologically active component(s) are not necessarily the same in different environments.

The results imply that special care should be taken with respect to the use of rhodanines stored in DMSO, which is widely used as a solvent in screening compounds both with isolated components and in cell/biological studies, including in pioneering studies on mirin.^6^ We suggest careful choice of solvent for rhodanine/rhodanine derived compounds, avoiding DMSO and using freshly prepared solutions where possible.

The 1,3-dithiolane and mixed disulfide products appear to be relatively stable under our reaction conditions, though it cannot be ruled out that other products (and intermediates) are formed, including under different conditions and when different types of enethiols are present.

A prior study has reported on the formation of 1,3-dithiolane [2-(4-methylpyridinium)-5-(4-pyridinium)-1,3-dithiolane-2,4-dicarboxylic acid] 9c from 9b on reaction in aqueous NaOH, with 9c being characterised by crystallography and NMR (with similar characteristic 1,3-dithiolane ^1^H resonances, but differently assigned ^13^C chemical shifts compared to our assignments, Fig. S33†).^20^ This study did not report on disulfide formation. Studies on rhodanine derived α-mercaptocinnamic acids have revealed formation of symmetric disulfides on reaction with iodine or benzoylperoxide,^21,22^ however, to our knowledge, none have described the selective formation of mixed disulfides, perhaps the most striking feature of our observations.

Further work is required to define the precise reasons for the differences in reactivity of the enethiols, including the apparent rate enhancement in DMSO, compared to other investigated solvents. DMSO is a S-containing and redox active solvent; however, these properties are not essential for the observed reactions as some reactions are observed in DMF, albeit to a lesser extent. We observed no evidence for DMSO derived adducts/intermediates, however, we cannot rule out their involvement. The differences may relate to enethiol-thiocarbonyl group tautomerisation which is reported to be affected by solvent polarity,^22–26^*e.g.* the enethiol form of thiodimedone (3-mercapto-5,5-dimethyl-2-cyclohexen-1-one) is preferred in nonpolar solvents and the thioketone form is preferred in polar solvents (ethanol, DMSO).^25^ Changes in the aryl substitution pattern would likely affect the tautomeric equilibrium,^17^ which may affect reaction rates and product distribution.

The mechanistic processes leading to the mixed disulfides and 1,3-dithiolanes are presently uncertain, but it is reasonable to propose a common intermediate is involved. It was previously proposed that 1,3-dithiolane formation occurs *via* an unusual “spontaneous double electrophilic addition” with Markovnikov type selectivity, initially involving reaction of two enethiols (Fig. 4a).^20^ This mechanism would predict similarly high levels of incorporation of ^2^H at both the H-11 and H-12 positions of the dithiolane product. However, on incubation of 1b in DMSO with ^2^H_2_O, we observed preferential incorporation at H-11 of 1c. Relatively low-level incorporation of ^2^H at H-12 of 1c was observed, likely due to thioketone–thioenol tautomerisation (Fig. S15 and S16†). On incubation of 2b in DMSO with ^2^H_2_O, we observed preferential incorporation of ^2^H at the H-2 position of the mixed disulfide 2d, with lower levels at the H-3 and H-12 positions of the mixed disulfide (Fig. S17 and S18†).

Consistent with the observations with ^2^H_2_O, we propose that the S of an enethiol(ate) tautomer reacts with the C of a thioketone tautomer to give an intermediate which can subsequently cyclise *via* 5-*endo-trig* Michael reaction (Fig. 4b).^27^ The latter step may be feasible due to the presence of the two sulfur atoms in the intermediate precursor to the 1,3-dithiolane. The S–S bond of the mixed disulfide can be formed by reaction of the S of enethiol(ate) tautomer with the S of a thioketone tautomer, with protonation at C-2 of the latter (Fig. 4).

Detailed kinetic studies are required to define the precise mechanisms involved and we cannot rule out the possibility that more than one mechanism operates for 1,3-dithiolane formation. The new mechanism proposed here, however, is attractive because of the different modes of reaction of an enethiol(ate) S with a common thioketone intermediate. The differences in 1,3-dithiolane and mixed disulfide product ratios observed with different substrates may, in part, reflect differences in enethiol(ate)/thioketone tautomer ratios and/or the rates of enethiol(ate) S reaction with the thioketone C or S atoms. Future work could involve studies with alkyl thioenols and non-enolisable thioketones.

The results suggest further work on the biological roles of enethiols including with respect to their potential to form mixed disulfides will be productive. Given that enethiols can be potent inhibitors of metallo-enzymes,^14,15^ the nature of their reactions with metal ions in biological systems is also of interest.

Enethiol-thiocarbonyl tautomerism is involved in biosynthesis of the antibiotic holomycin^17^ and coenzyme A.^18,28^ The oxidation of cysteine residues to vinyl thioethers likely *via* enethiols has been shown to occur during biosynthesis of the lanthipeptides and related post-translationally modified peptides,^19^ and enethiol related products can be formed in catalysis by isopenicillin N synthase (Fig. 1c).^16^ Our results suggest that certain mixed disulfides may be particularly prone to being formed from enethiols; however characterising mixed disulfides in cells is technically challenging. Thus, studies with isolated molecules as exemplified here and the availability of standards are important in supporting biological research.

## Author contributions

J. J. A. G. K. manuscript writing, reviewing and editing, data collection, product characterisation; D. Z. preparation of enethiols, initial discovery of reactivity. T. D. W. C. manuscript writing, reviewing and editing, supervision; C. J. S. manuscript writing, conceptualisation, reviewing and editing, supervision.

## Conflicts of interest

There are no conflicts to declare.

## Supplementary Material

MD-015-D4MD00157E-s001
