# Supplementary material for: Rhodanine derived enethiols react to give 1,3-dithiolanes and mixed disulfides
Source: RSC Med Chem. 2024 Jun 5;15(7):2305–9. doi: 10.1039/d4md00157e (PMC11253852; doi:10.1039/d4md00157e)
Supplement: MD-015-D4MD00157E-s001 [file MD-015-D4MD00157E-s001.pdf]

Electronic Supplementary Material (ESI) for RSC Medicinal Chemistry.
This journal is © The Royal Society of Chemistry 2024

Rhodanine Derived Enethiols React to Give 1,3-Dithiolanes and Mixed Disulfides

Supplementary Information

Jos J. A. G. Kamps[1,2], Dong Zhang[1], Timothy D. W. Claridge[1,3] and Christopher J. Schofield.[1*]

[1] Chemistry Research Laboratory, Department of Chemistry and the Ineos Oxford Institute for Antimicrobial Research, University of Oxford, Oxford OX1 3TA, United Kingdom.

[2]Current address: Diamond Light Source, Diamond House, Rutherford Appleton Laboratories, Didcot, United Kingdom.

[3] Current address: Exscientia, The Schrödinger Building, Oxford Science Park, Oxford OX4 4GE, United Kingdom.

Contents

Contents 2

.... 3

General Experimental Conditions 36

NMR Characterisation of products 37

(2*R*,4*R*,5*R*)-2-Benzyl-5-phenyl-1,3-dithiolane-2,4-dicarboxylic acid (1c) 37

(*Z*)-2-((1-Carboxy-2-phenylethyl)disulfaneyl)-3-phenylacrylic acid (1d) 37

(2*R*,4*R*,5*R*)-5-Phenyl-2-(phenylmethyl-d)-1,3-dithiolane-2,4-dicarboxylic-4-d acid (1c-DD) 37

(*Z*)-2-((1-Carboxy-2-(2-chloro-6-fluorophenyl)ethyl)disulfaneyl)-3-(2-chloro-6-fluorophenyl)acrylic acid (2d) 38

(2*R*,4*R*,5*R*)-2-(2-Chloro-6-fluorobenzyl)-5-(2-chloro-6-fluorophenyl)-1,3-dithiolane-2,4-dicarboxylic acid (2c) 38

(*Z*)-2-((1-Carboxy-2-(2,6-difluorophenyl)ethyl)disulfaneyl)-3-(2,6-difluorophenyl)acrylic acid (4d) 39

(*Z*)-2-((1-Carboxy-2-(2,6-dichlorophenyl)ethyl)disulfaneyl)-3-(2,6-dichlorophenyl)acrylic acid (5d) 40

(*Z*)-2-((1-Carboxy-2-(2,6-dimethylphenyl)ethyl)disulfaneyl)-3-(2,6-dimethylphenyl)acrylic acid (6d) 40

(2*R*,4*R*,5*R*)-2-(4-Hydroxybenzyl)-5-(4-hydroxyphenyl)-1,3-dithiolane-2,4-dicarboxylic acid (7c) 40

(*Z*)-2-((1-Carboxy-2-(4-hydroxyphenyl)ethyl)disulfaneyl)-3-(4-hydroxyphenyl)acrylic acid (7d) 41

(2*R*,4*R*,5*R*)-2-(4-Methoxybenzyl)-5-(4-methoxyphenyl)-1,3-dithiolane-2,4-dicarboxylic acid (8c) .. 41

(*Z*)-2-((1-Carboxy-2-(4-methoxyphenyl)ethyl)disulfaneyl)-3-(4-methoxyphenyl)acrylic acid (8d) ... 41

References 42

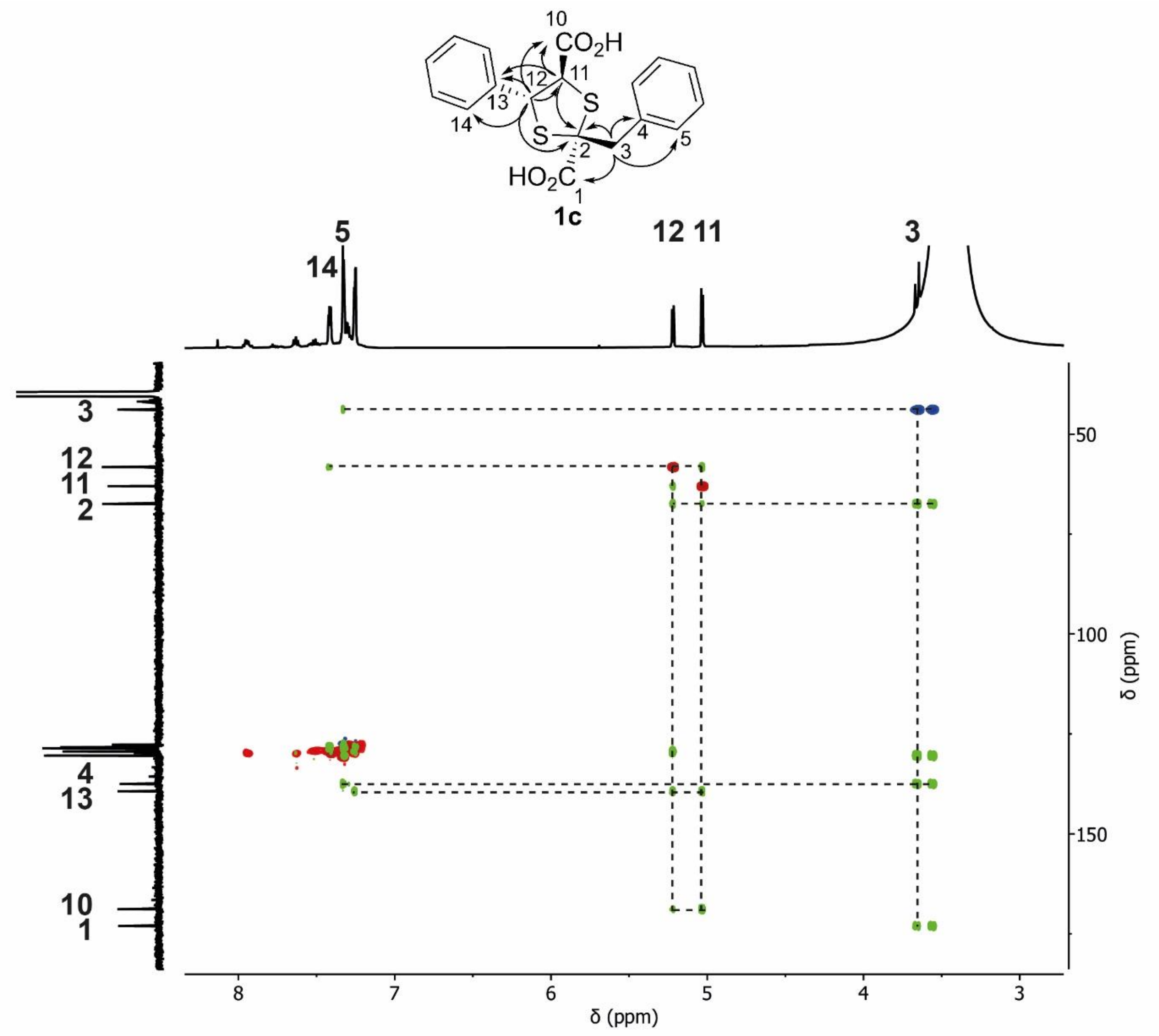

Figure S1: ^1H-^{13}C HSQC and HMBC NMR (700, 176 MHz, 298 K) characterisation of **1c**, the major observed product when **1b** (5 mM) reacts in DMSO-d_6 for 10 hours at room temperature. Cross peaks of the phase sensitive HSQC are in blue and red. Green peaks represent HMBC through bond correlations. Dashed lines represent through bond HMBC couplings, indicated by arrows in the structure of **1c**.

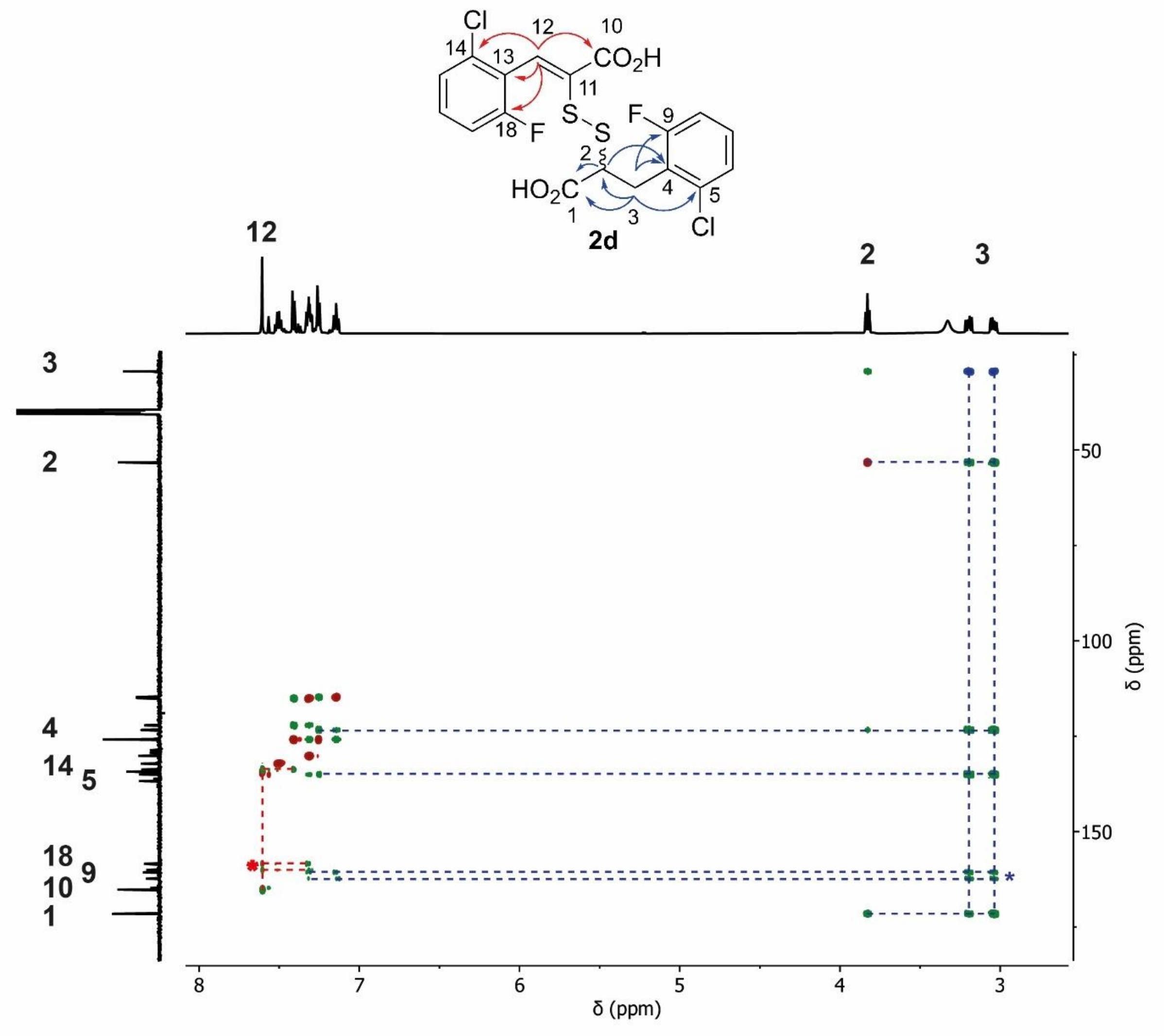

Figure S2: ^{1}H-^{13}C HSQC and HMBC NMR (700 MHz, 298 K) characterisation of **2d**, the observed product when **2b** (50 mM) reacts in DMSO-d_6 for 2 hours at room temperature. Cross peaks of the phase sensitive HSQC are in blue and red. Green peaks represent HMBC through bond correlations. Dashed lines represent through bond HMBC couplings, which are labelled by arrows in the structure of **2d**. The dashed lines and arrows are colour coded to match the couplings they represent. *Splitting of the ^{13}C signal as a result of ^{19}F coupling.

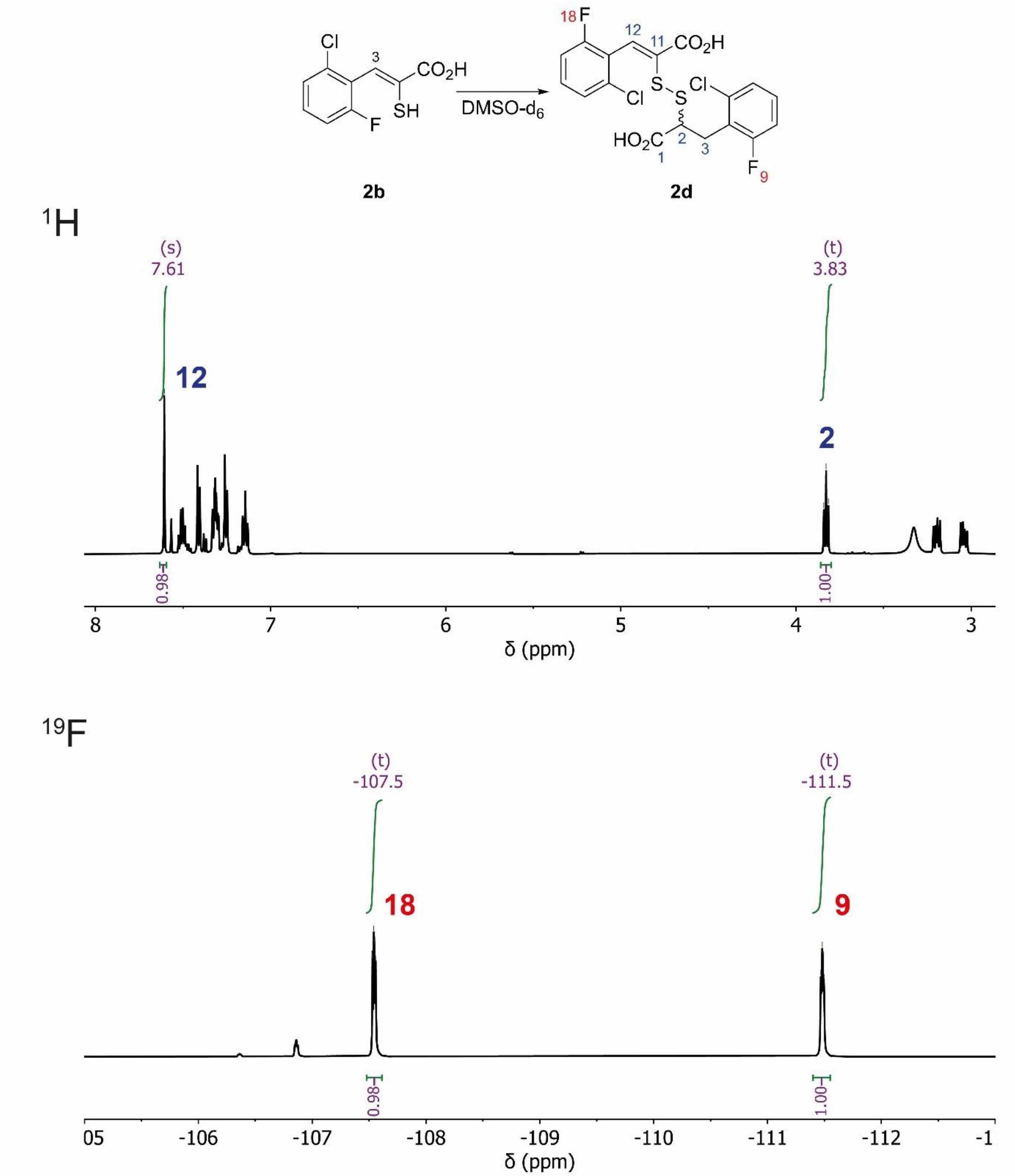

Figure S3: ^{1}H NMR (600 MHz, 298 K) and ^{19}F NMR (565 MHz, 298 K) analyses of **2b** (5 mM) in DMSO-d_6 after reacting for 2 hours at room temperature to give **2d**. Comparison of the integrals for H-2 and H-12, and F-9 and F-18 indicates a near 1 : 1 stoichiometry of the two 'components' forming the mixed disulfide **2d**.

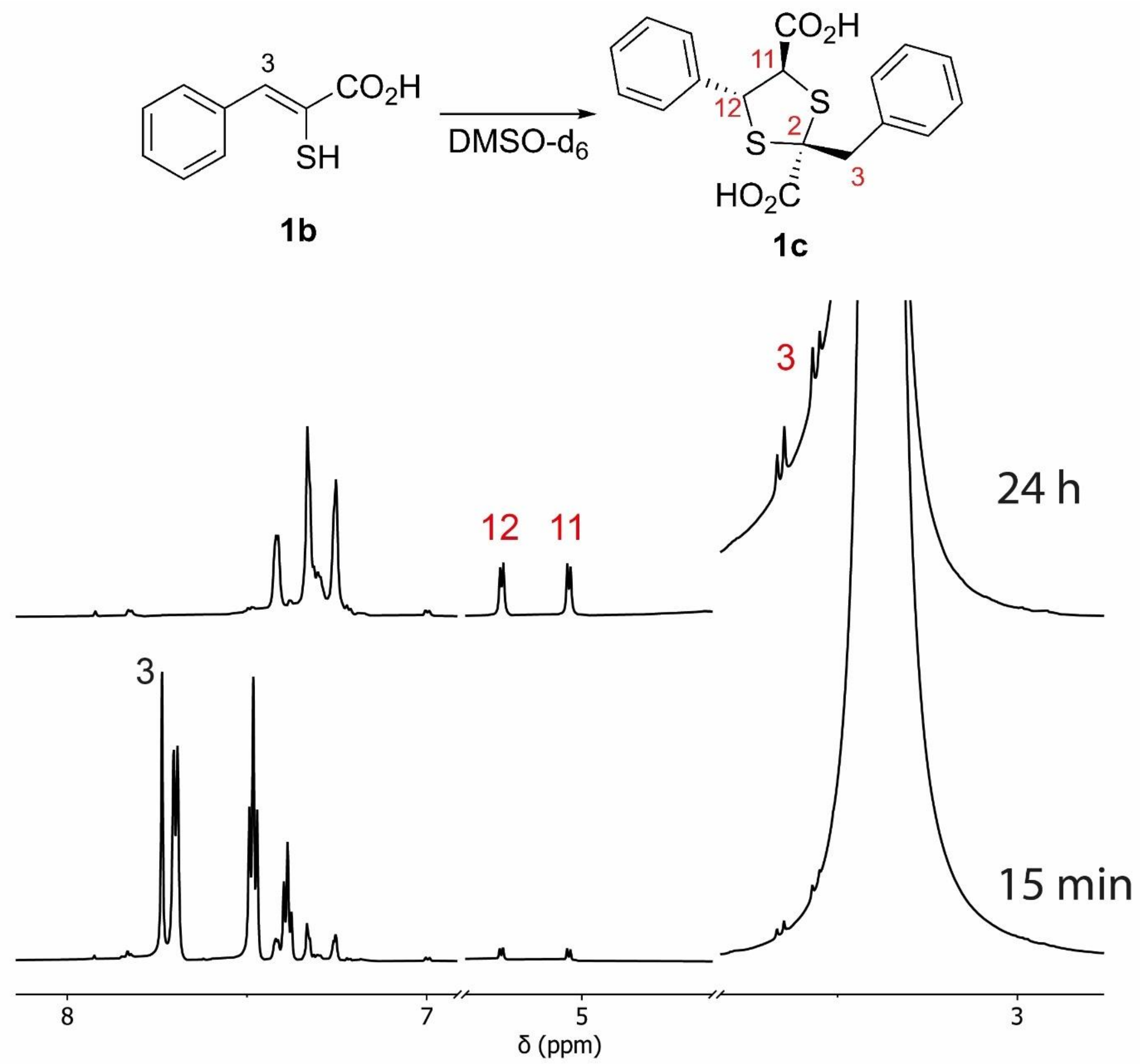

Figure S4: ^{1}H NMR (700 MHz, 298 K) analysis of the reaction of **1b** (5 mM) to form **1c** in DMSO-d$_6$ under anaerobic conditions, after 15 minutes, and 24 hours (room temperature).

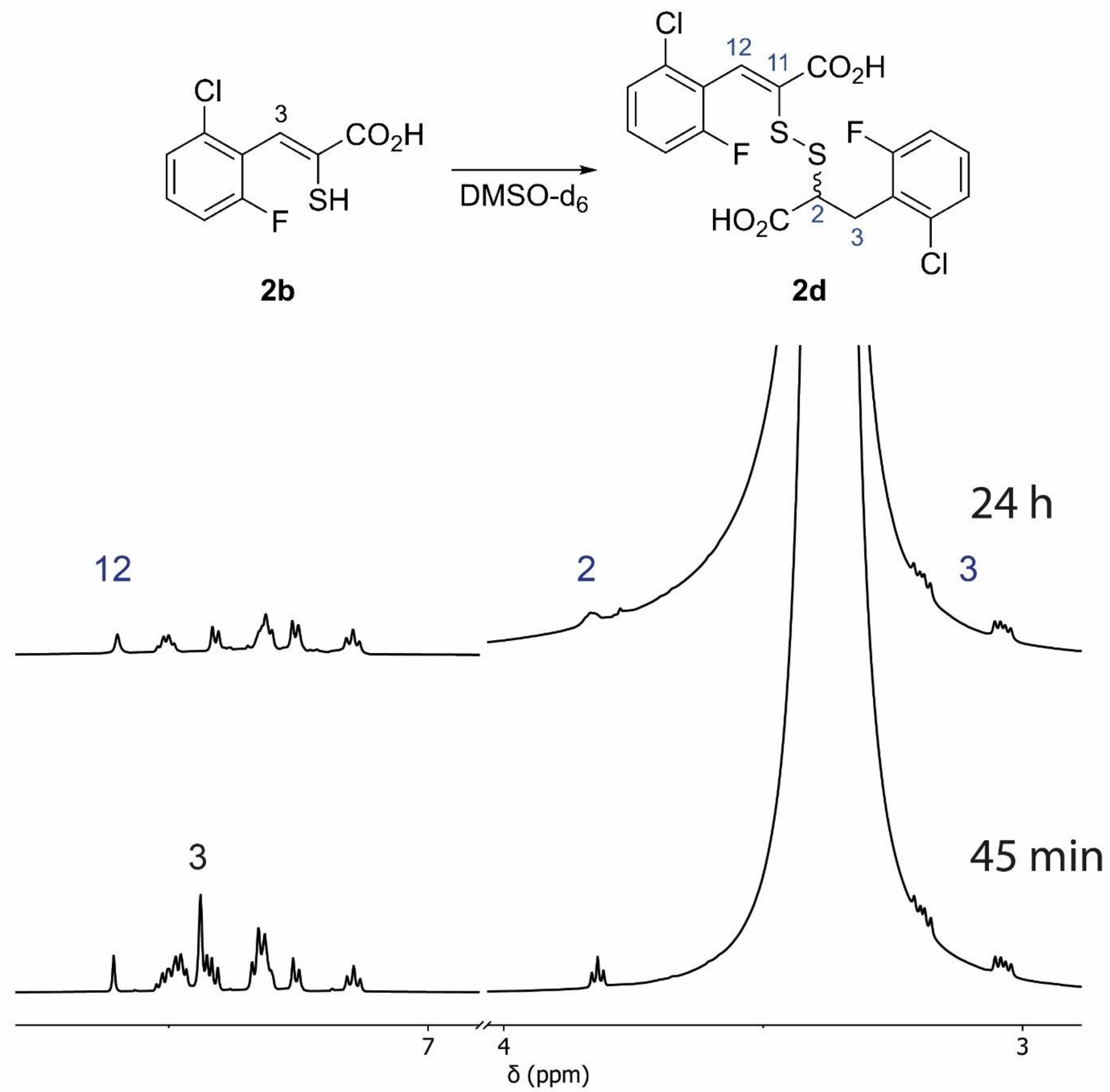

Figure S5: ^{1}H NMR (700 MHz, 298 K) analysis of the reaction of **2b** (5 mM) to form **2d** in DMSO-d_6 under anaerobic conditions, after 45 minutes and 24 hours (room temperature).

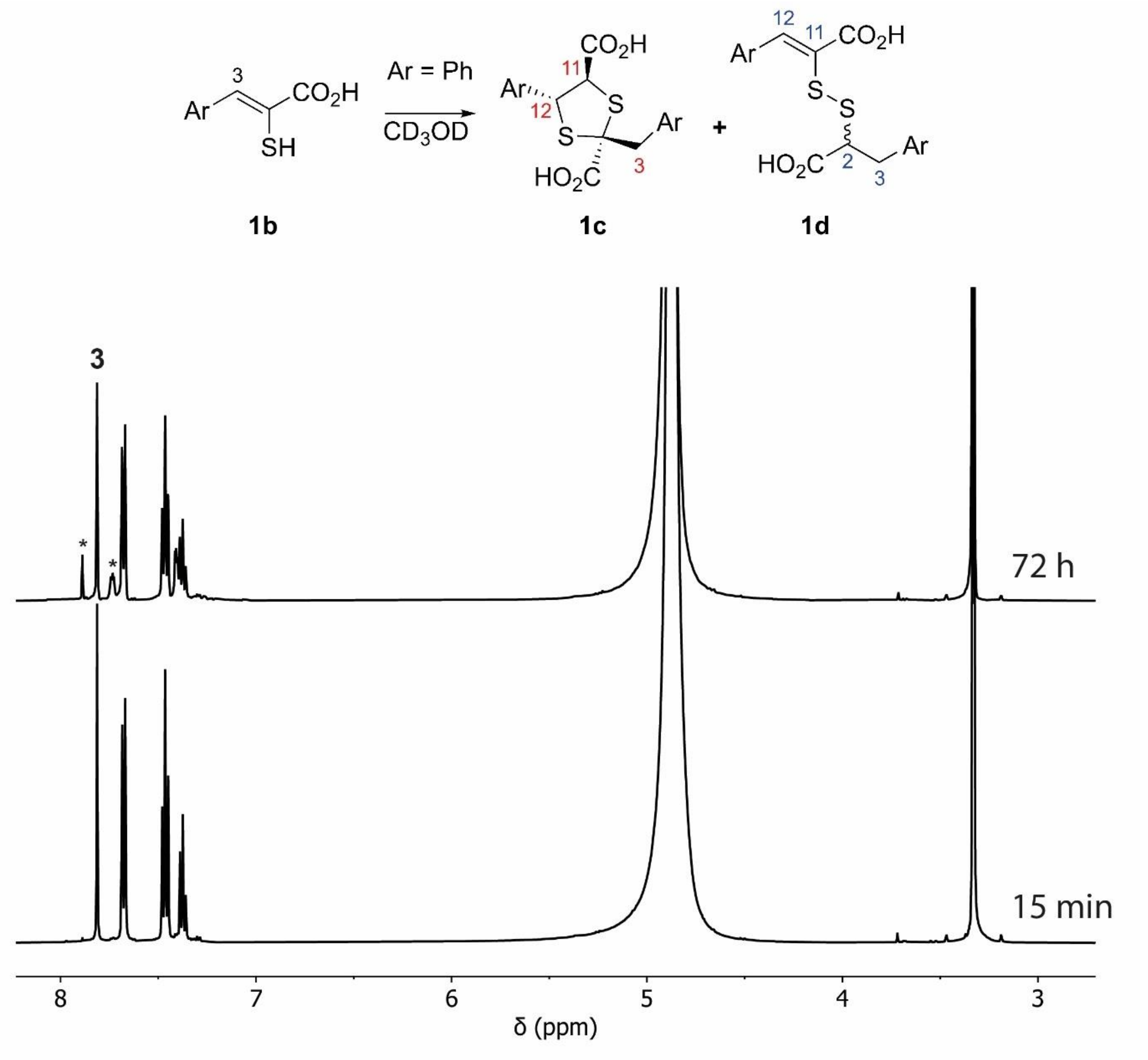

Figure S6: ^{1}H NMR (600 MHz, 298 K) analysis of the reaction of **1b** (5 mM) in methanol-d$_4$ for 72 hours at room temperature. No evidence for substantial formation of **1c** or **1d** was observed. *Indicates potential formation of the symmetric unsaturated disulfide.

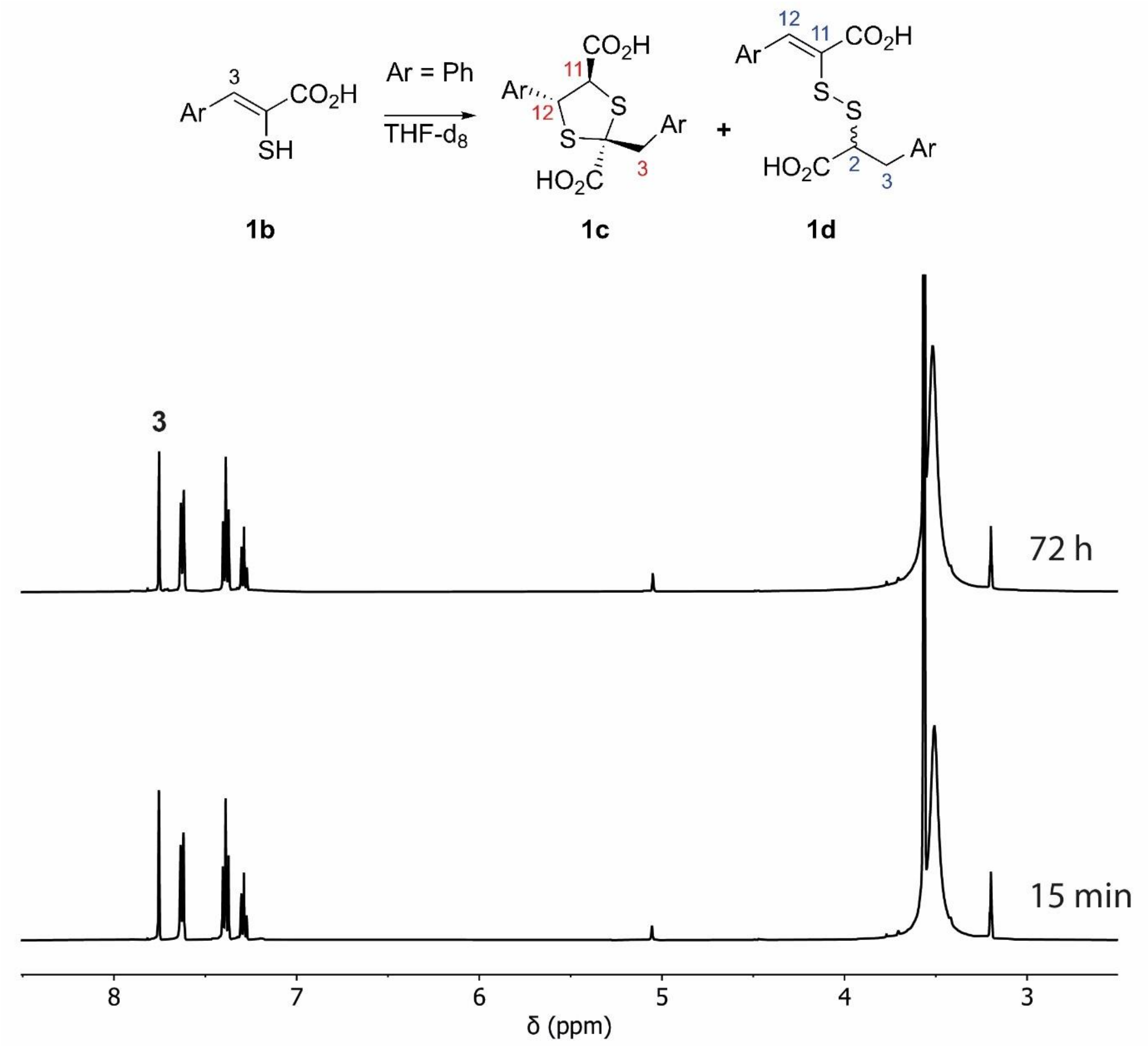

Figure S7: ^{1}H NMR (600 MHz, 298 K) analysis of the reaction of **1b** (5 mM) in THF-d_8 for 72 hours at room temperature. No evidence for substantial formation of **1c** or **1d** was observed.

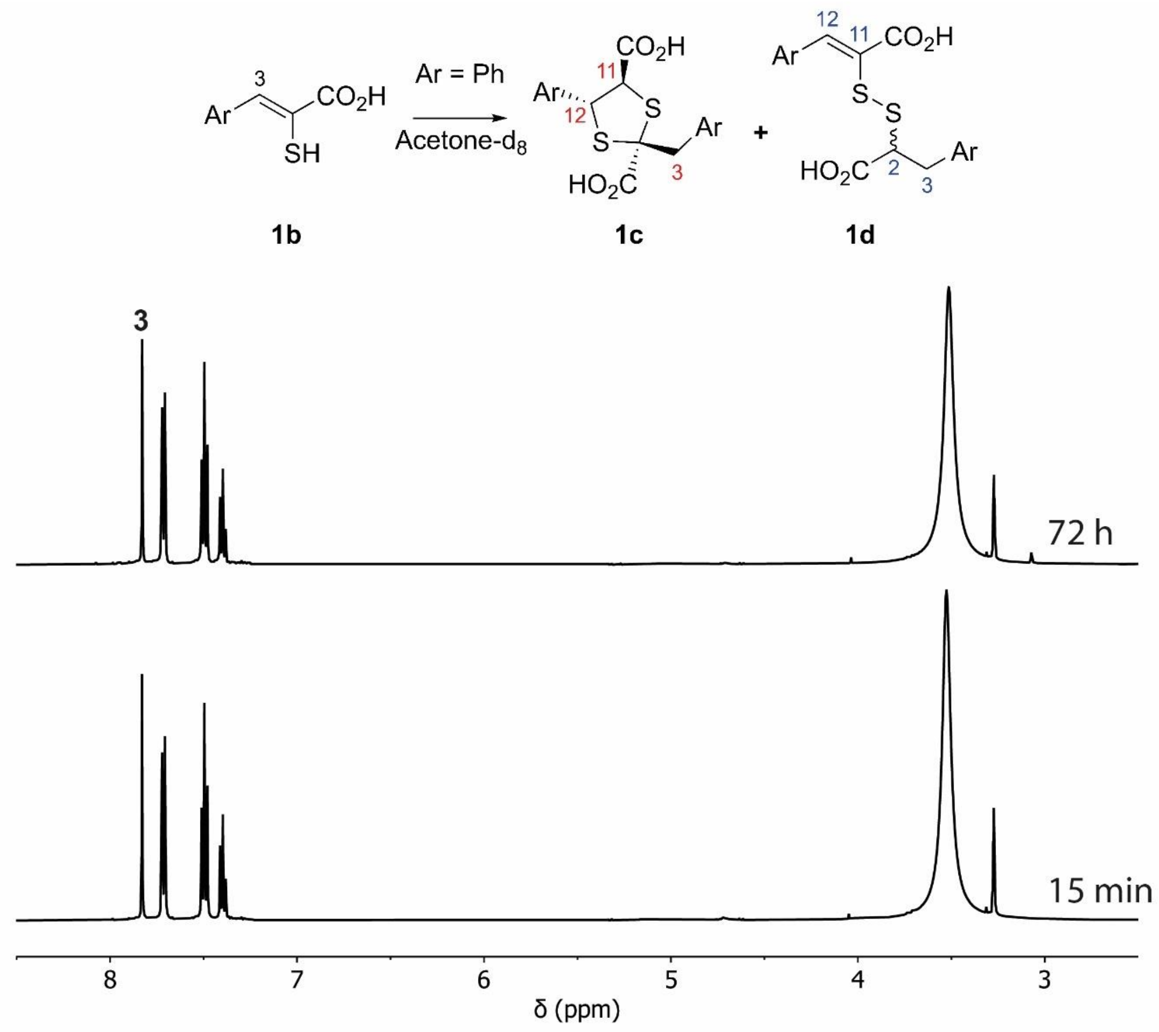

Figure S8: ^{1}H NMR (600 MHz, 298 K) analysis of the reaction of **1b** (5 mM) in acetone-d_6 for 72 hours at room temperature. No evidence for substantial formation of **1c** or **1d** was observed.

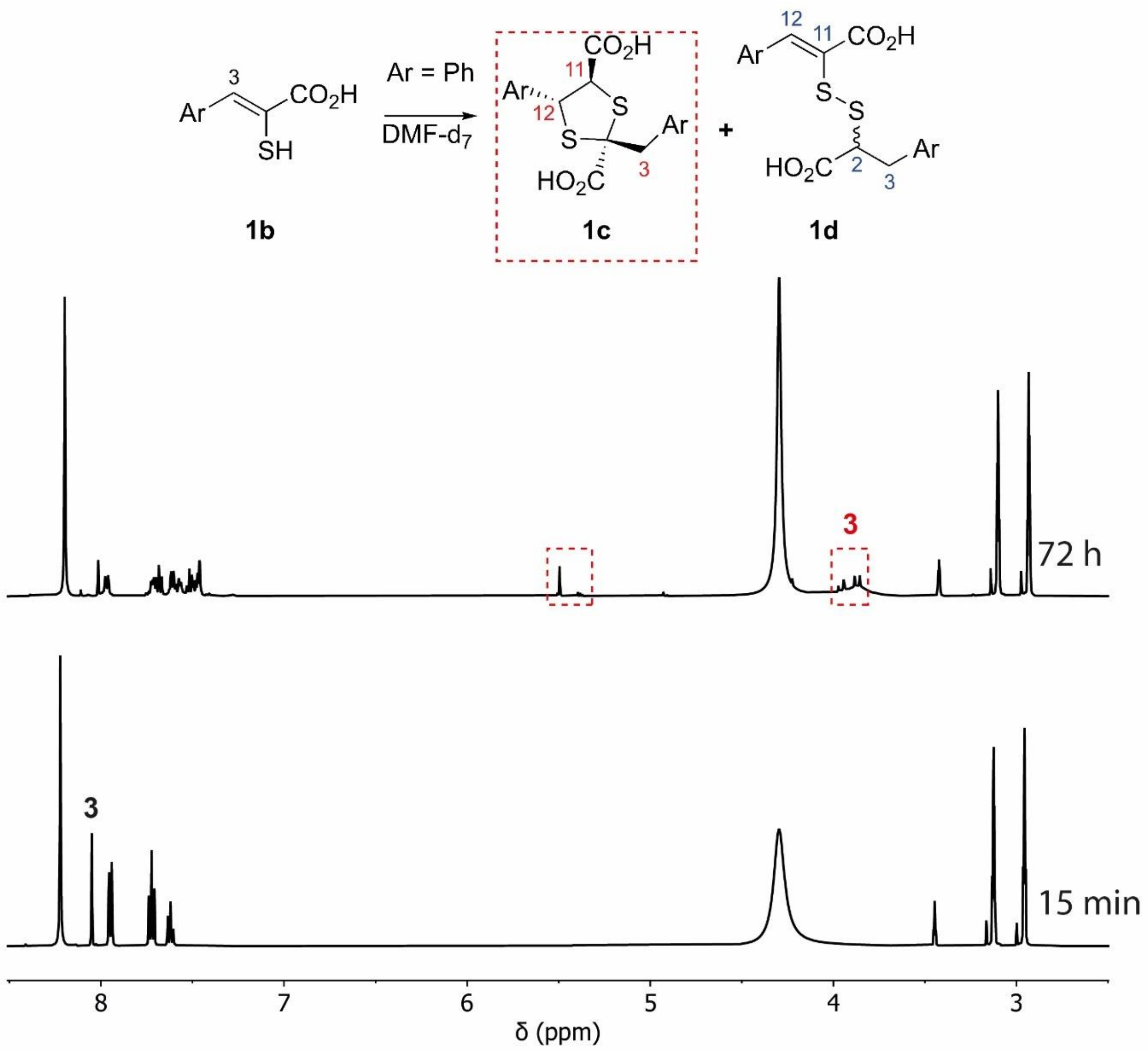

Figure S9: ^{1}H NMR (600 MHz, 298 K) analysis of the reaction of **1b** (5 mM) in DMF-d_7 for 72 hours at room temperature. Evidence for formation of **1c** as indicated by the peaks (red box), albeit incomplete, was observed after 72 hours.

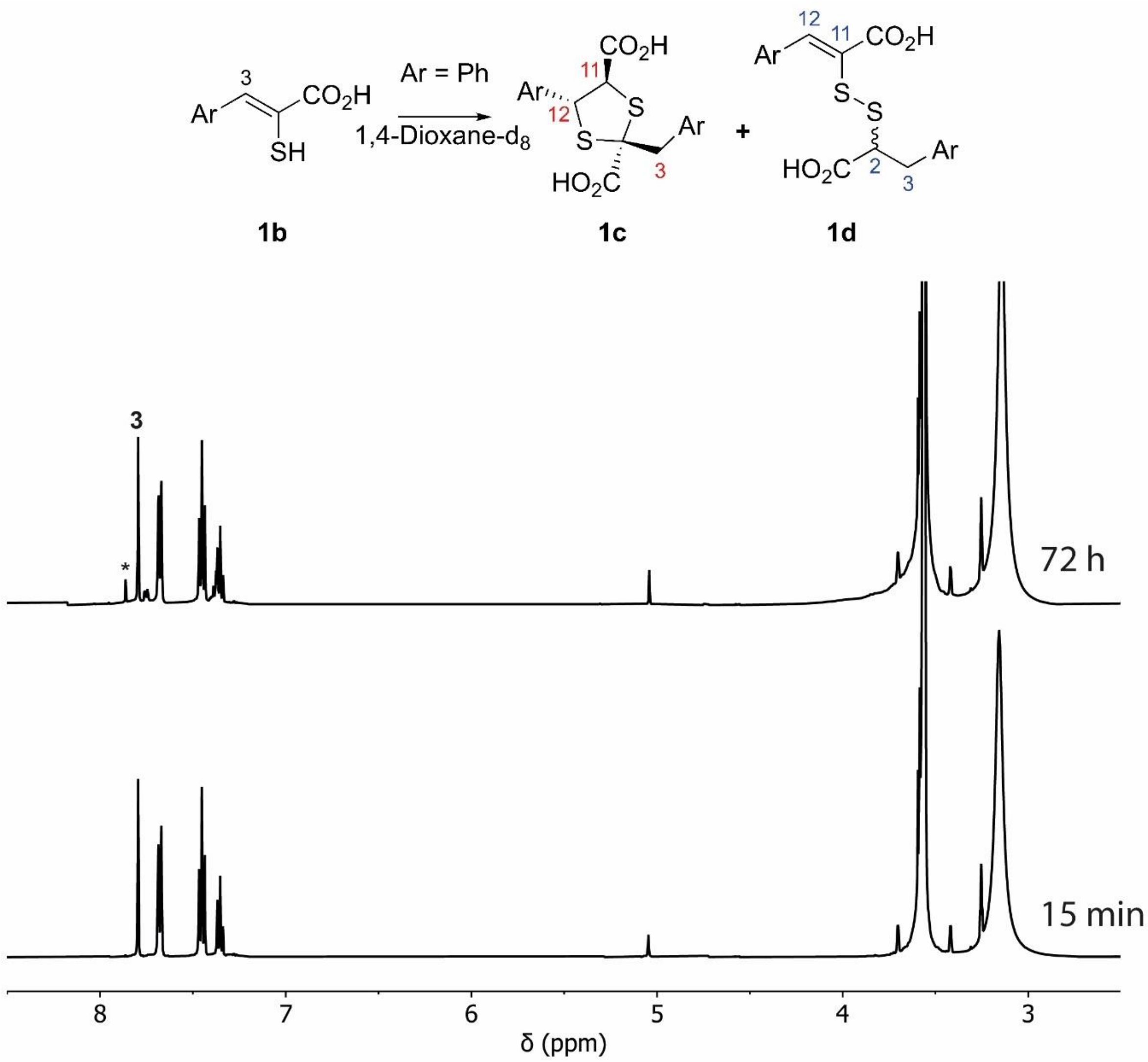

Figure S10: ^{1}H NMR (600 MHz, 298 K) analysis of the reaction of **1b** (5 mM) in 1,4-dioxane-d$_8$ for 72 hours at room temperature. No evidence for substantive formation of **1c** or **1d** was observed. *Indicates potential formation of a symmetric unsaturated disulfide.

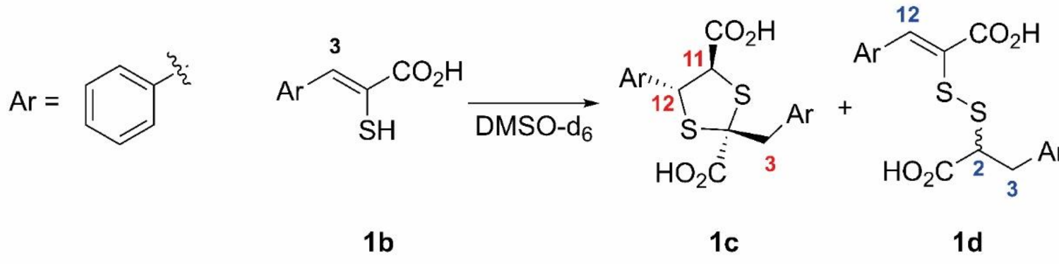

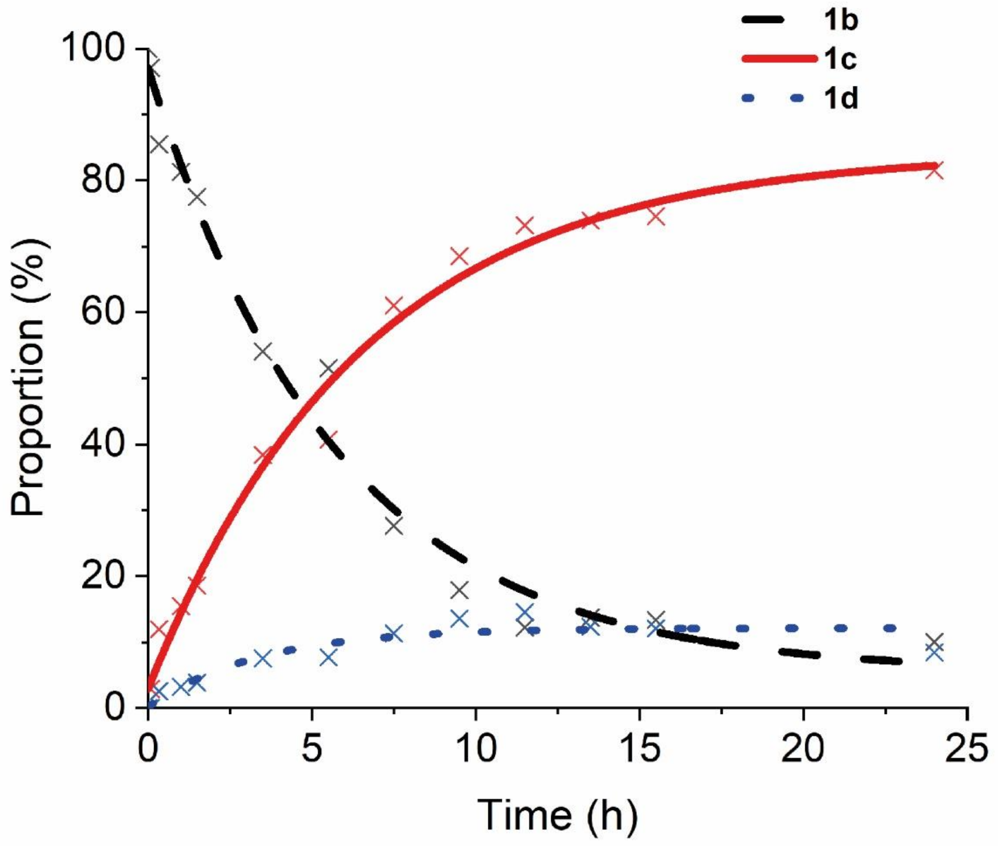

Figure S11: Reaction of **1b** (5 mM) in DMSO-d_6 at room temperature monitored over time, using ^{1}H NMR (500 MHz, 298 K). The plot is derived from relative integrals of characteristic non-overlapping resonances for the precursor **1b** (H-3) and products **1c** (H-12) and **1d** (H-2), expressed as a percentage of the total. Data were fitted using non-linear curve fits using OriginPro (2021b 9.8.5.212) with R^2 being 0.983 (enethiol, **1b**), 0.987 (1,3-dithiolane, **1c**), and 0.897 (mixed disulfide, **1d**).

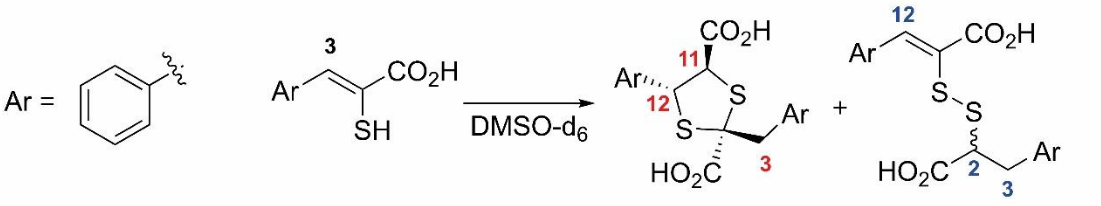

1b **1c** **1d**

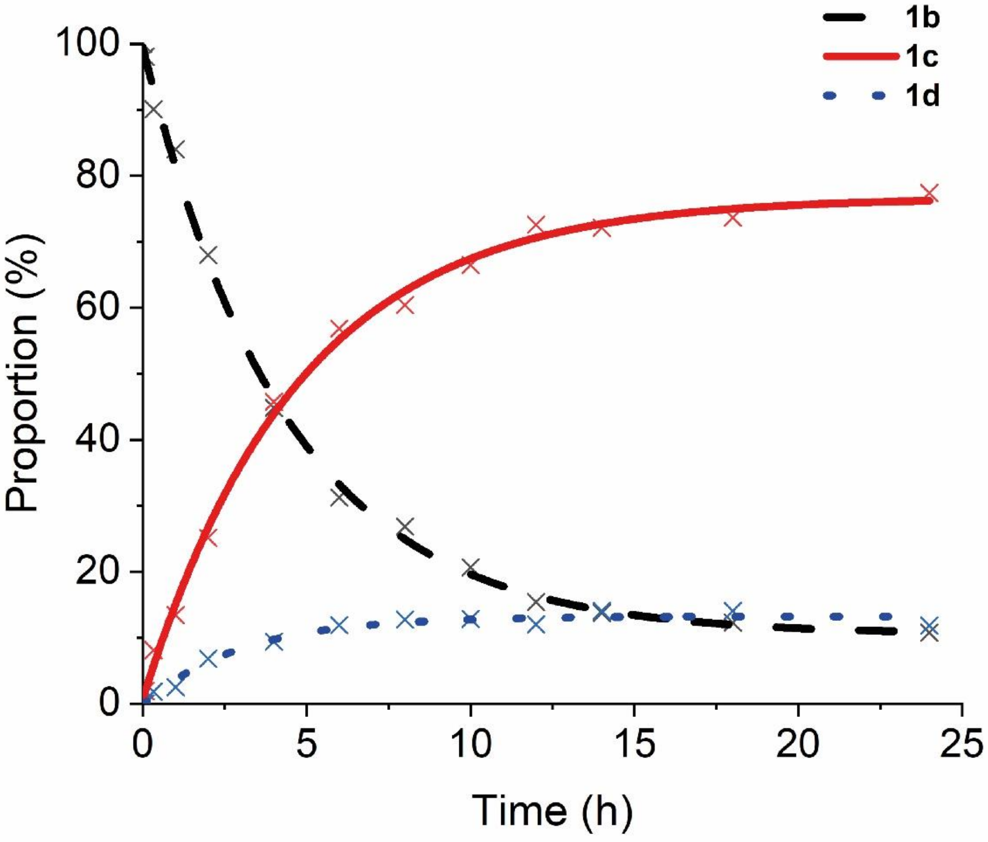

Figure S12: Reaction of **1b** (10 mM) in DMSO-d$_6$ at room temperature monitored over time, using ^{1}H NMR (500 MHz, 298 K). The plot is derived from relative integrals of characteristic non-overlapping resonances for the precursor **1b** (H-3) and products **1c** (H-12) and **1d** (H-2), expressed as a percentage of the total. Data were fitted using non-linear curve fits using OriginPro (2021b 9.8.5.212) with R^2 being 0.998 (enethiol, **1b**), 0.997 (1,3-dithiolane, **1c**), and 0.981 (mixed disulfide, **1d**).

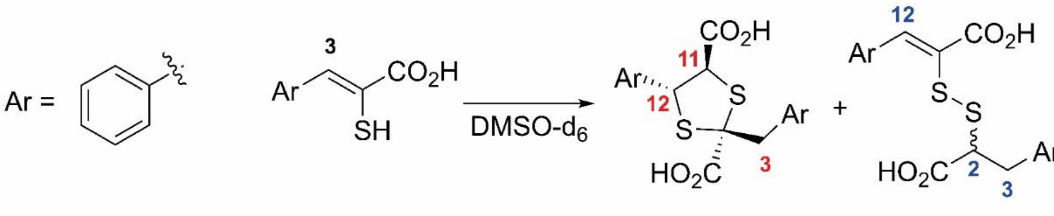

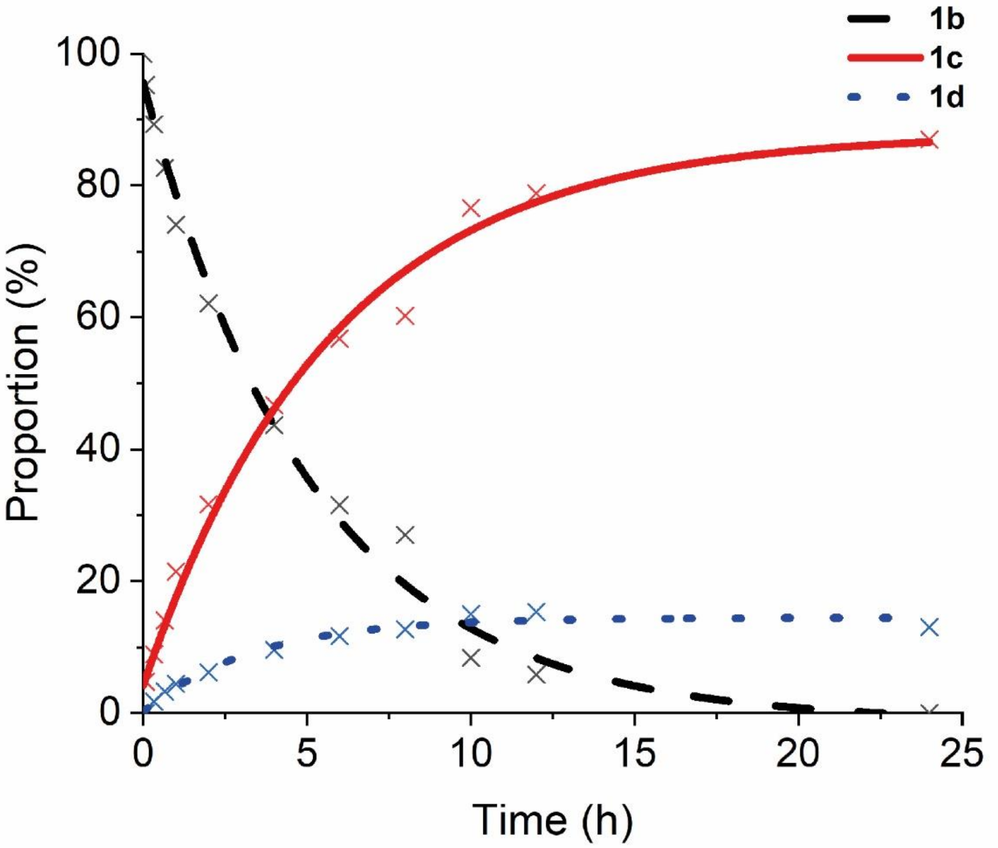

Figure S13: Reaction of **1b** (50 mM) in DMSO-d_6 at room temperature monitored over time, using ^{1}H NMR (500 MHz, 298 K). The plot is derived from relative integrals of characteristic non-overlapping resonances for the precursor **1b** (H-3) and products **1c** (H-12) and **1d** (H-2) and expressed as percentage of the total. Data were fitted using non-linear curve fits using OriginPro (2021b 9.8.5.212) with R^2 being 0.991 (enethiol, **1b**), 0.990 (1,3-dithiolane, **1c**), and 0.981 (mixed disulfide, **1d**).

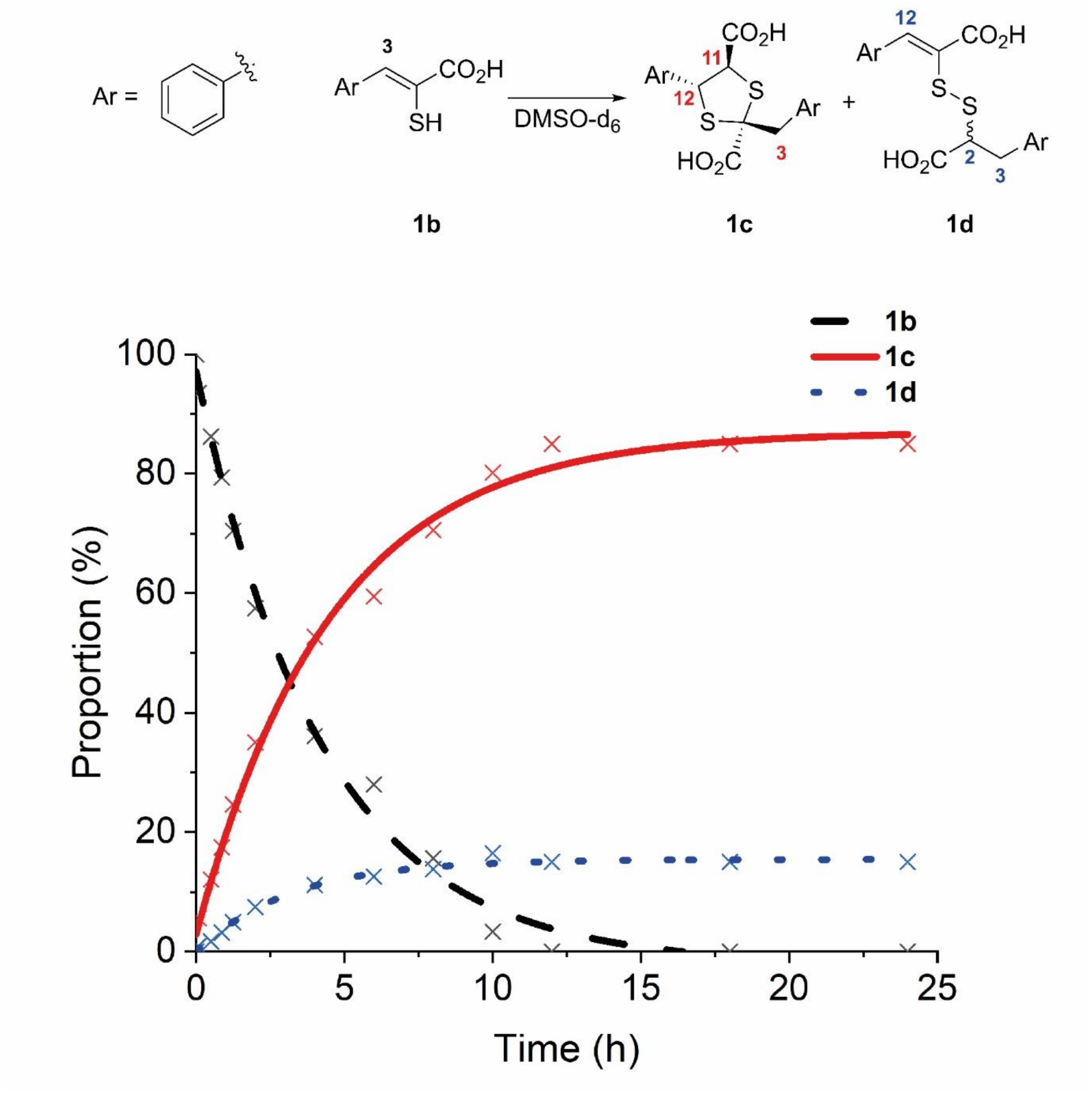

Figure S14: Reaction of **1b** (100 mM) in DMSO-d$_6$ at room temperature monitored over time, using ^{1}H NMR (500 MHz, 298 K). The plot is derived from relative integrals of characteristic non-overlapping resonances for the precursor **1b** (H-3) and products **1c** (H-12) and **1d** (H-2) and expressed as percentage of the total. Data were fitted using non-linear curve fits using OriginPro (2021b 9.8.5.212) with R^2 being 0.994 (**1b**), 0.994 (1,3-dithiolane, **1c**), and 0.990 (mixed disulfide, **1d**).

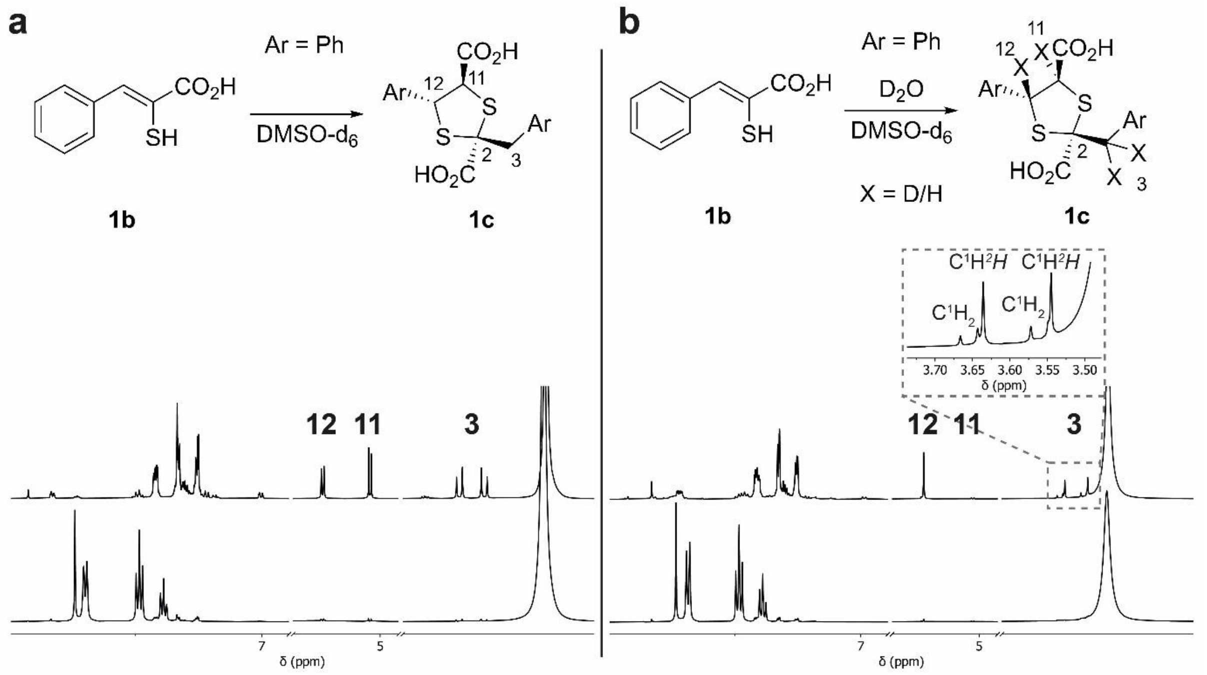

Figure S15: ^{1}H NMR (600 MHz, 298 K) experiments to investigate potential incorporation of ^{2}H from $^{2}H_2O$ into **1c**. a) Reaction of **1b** (5 mM) in DMSO-d_6 (450 µL) performed in the presence of H_2O (20 µL) at room temperature. b) Reaction of **1b** (5 mM) in DMSO-d_6 (450 µL) in the presence of $^{2}H_2O$ (20 µL) at room temperature.

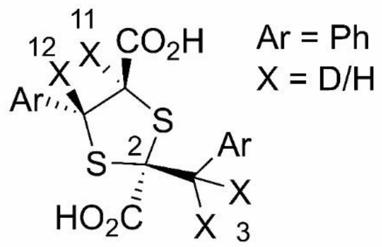

1c

position	^{1}H (%)	^{2}H (%)	$C^{1}H_2$ (%)	$C^{1}H^{2}H$ (%)	$C^{2}H_2$ (%)
3	-	-	16	35	49
11	0	100	-	-	-
12	89	11	-	-	-

Figure S16: Incorporation of ^{2}H from $^{2}H_2O$ into **1c** based on relative ^{1}H NMR integrals. Percentages are derived using integrals from the ^{1}H NMR (600 MHz, 298 K) spectra, comparing the resonance corresponding to the indicated protons and non-overlapping aromatic resonances.

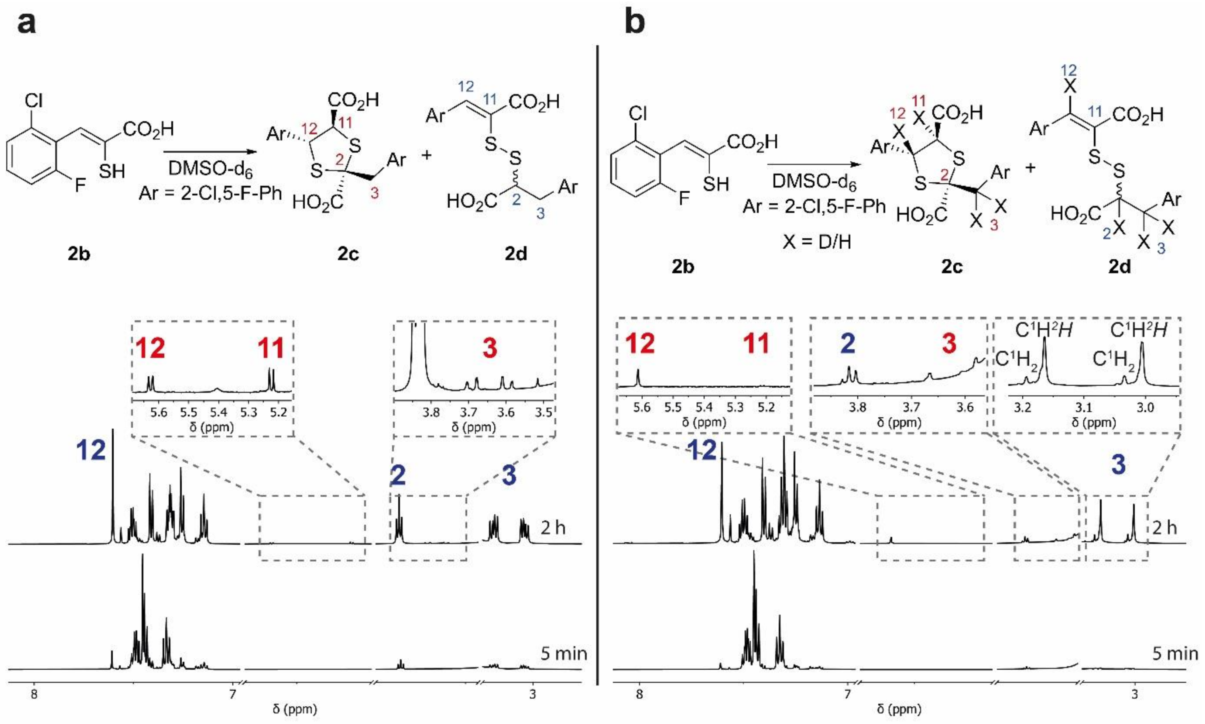

Figure S17: ^{1}H NMR (600 MHz, 298 K) experiments to investigate potential incorporation of ^{2}H from ^2H_2O into **2c** and **2d**. a) Reaction of **2b** (5 mM) in DMSO-d$_6$ (450 µL) in the presence of H_2O (20 µL). b) Reaction of **2b** (5 mM) in DMSO-d$_6$ (450 µL) in the presence of ^2H_2O (20 µL). The intensity of the **2d** CH-2 resonance (δ_H 3.82 ppm) in the ^2H_2O supplemented sample does not change appreciably over the duration of the experiment; the **2d** (CH_2-3) resonances (δ_H 3.17 and 3.01 ppm) increase in intensity over the duration of the experiment, however, appear as two singlets.

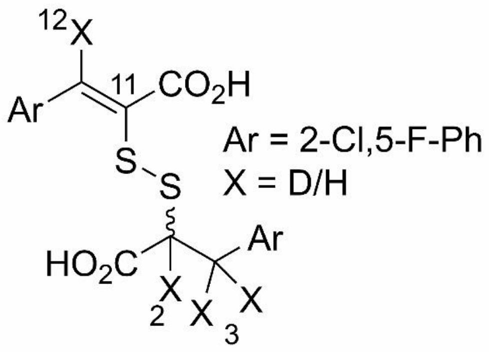

2d

position	^1H (%)	^2H (%)	C^1H_2 (%)	C^1H^2H (%)	C^2H_2 (%)
2	0	100	-	-	-
3	-	-	15	40	45
12	88	12	-	-	-

Figure S18: Incorporation of ^2H into **2d** based on relative ^1H NMR integrals. Percentages are derived using relative integrals from the ^1H NMR (600 MHz, 298 K) spectra, comparing the resonance corresponding to the indicated protons and a non-overlapping aromatic resonance.

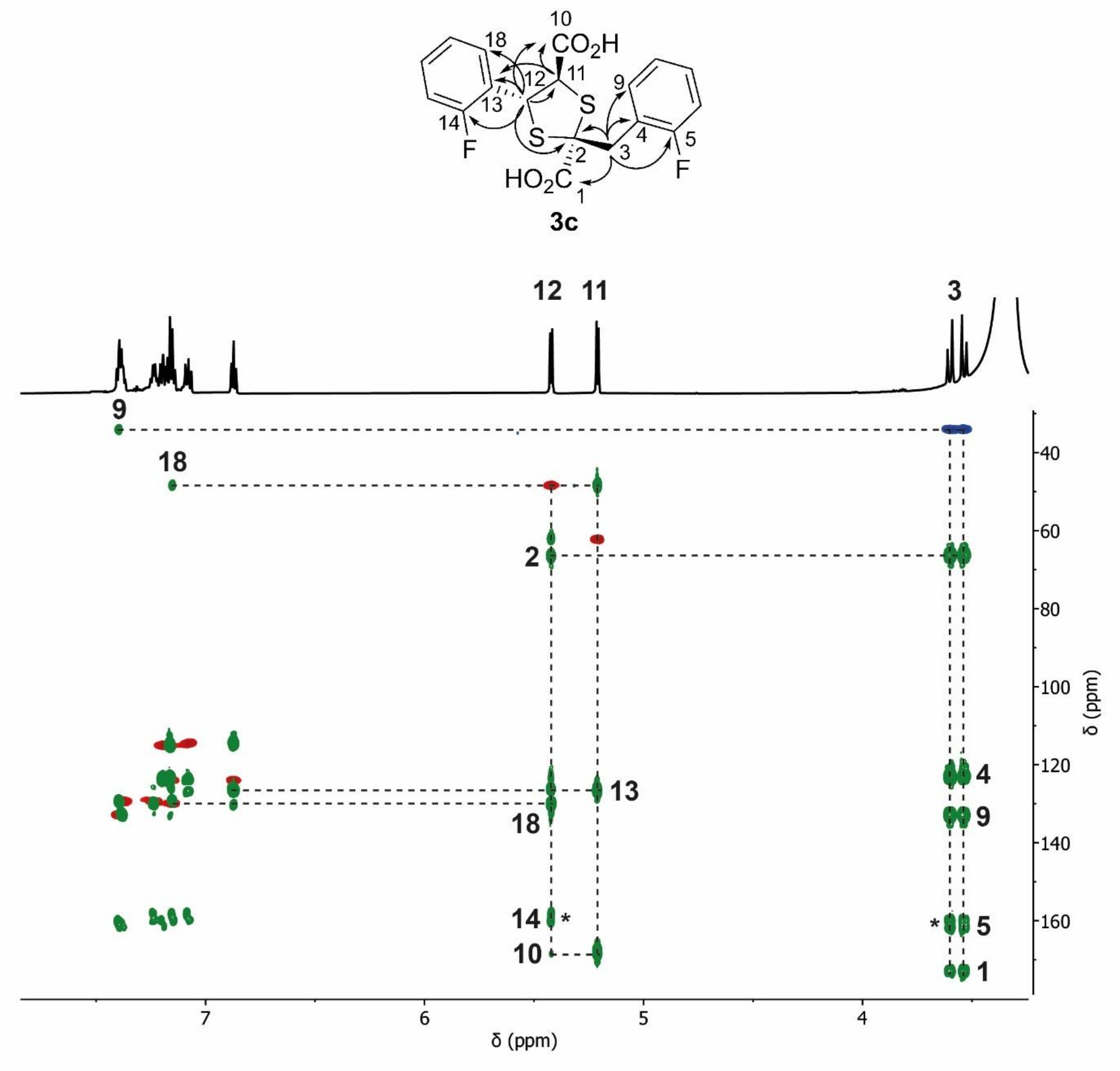

Figure S19: ^{1}H-^{13}C HSQC and HMBC NMR (700, 176 MHz, 298 K) characterisation of **3c** the major product formed when **3b** (5 mM) reacts in DMSO-d$_6$ for 1 hour at room temperature. Cross peaks of the phase sensitive HSQC are in blue and red. Green peaks represent HMBC through bond correlations. Dashed lines represent through bond HMBC couplings, which are labelled by arrows in the structure. The arrows represent couplings and correspond to the dashed lines which highlight couplings used to assign **3c**. *^{13}C signal split as a result of ^{19}F coupling.

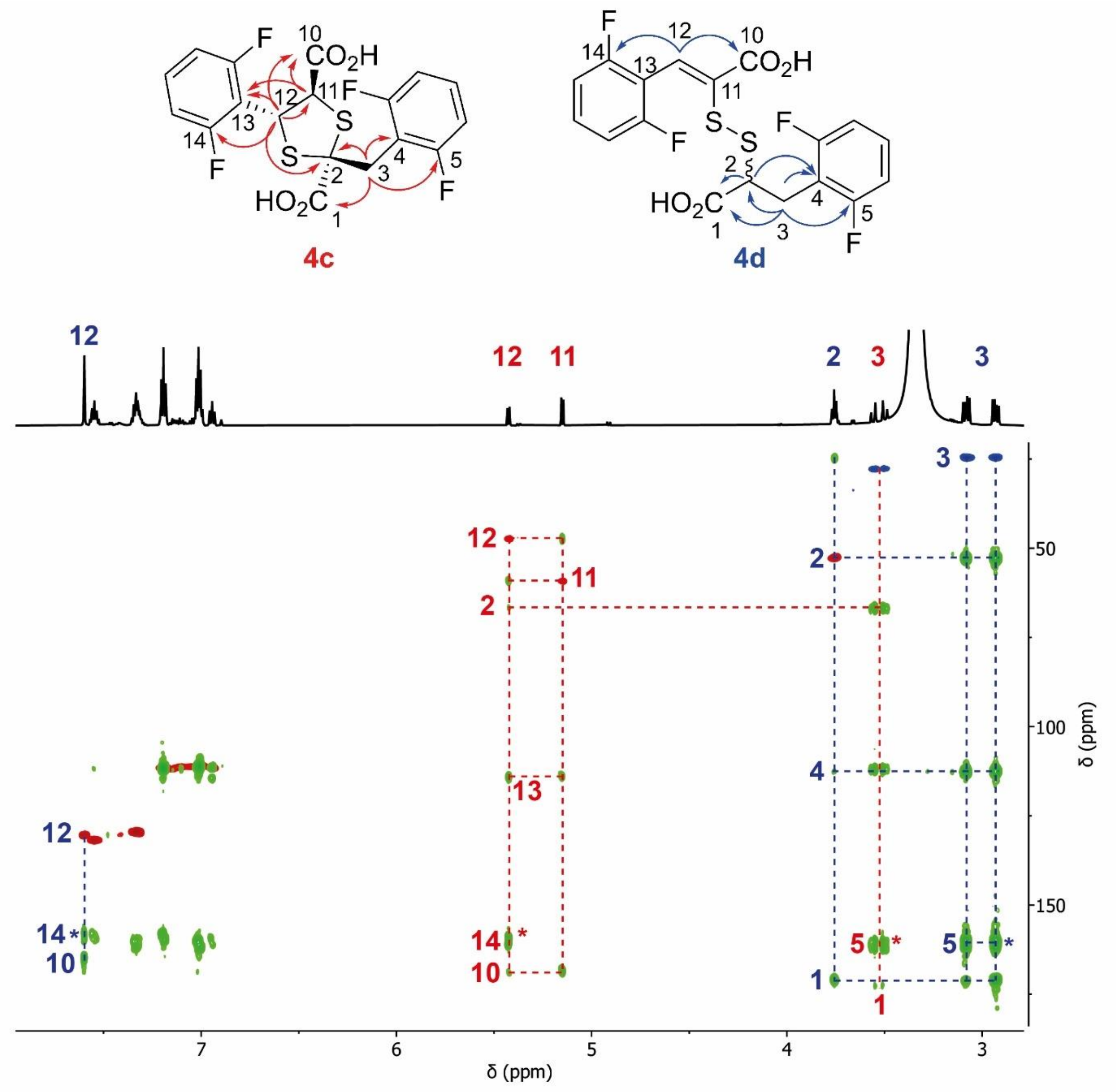

Figure S20: ^{1}H-^{13}C HSQC and HMBC NMR (700, 176 MHz, 298 K) characterisation of **4c** and **4d**, the major products formed when **4b** (5 mM) reacts in DMSO-d$_6$ for 20 minutes at room temperature. Cross peaks of the phase sensitive HSQC are in blue and red. Green peaks represent HMBC through bond correlations. Dashed lines represent through bond HMBC couplings, which are labelled by arrows in the structure. The arrows represent couplings and correspond to the colour matched dashed lines which highlight couplings used to assign **4c** and **4d**. *^{13}C signal split as a result of ^{19}F coupling.

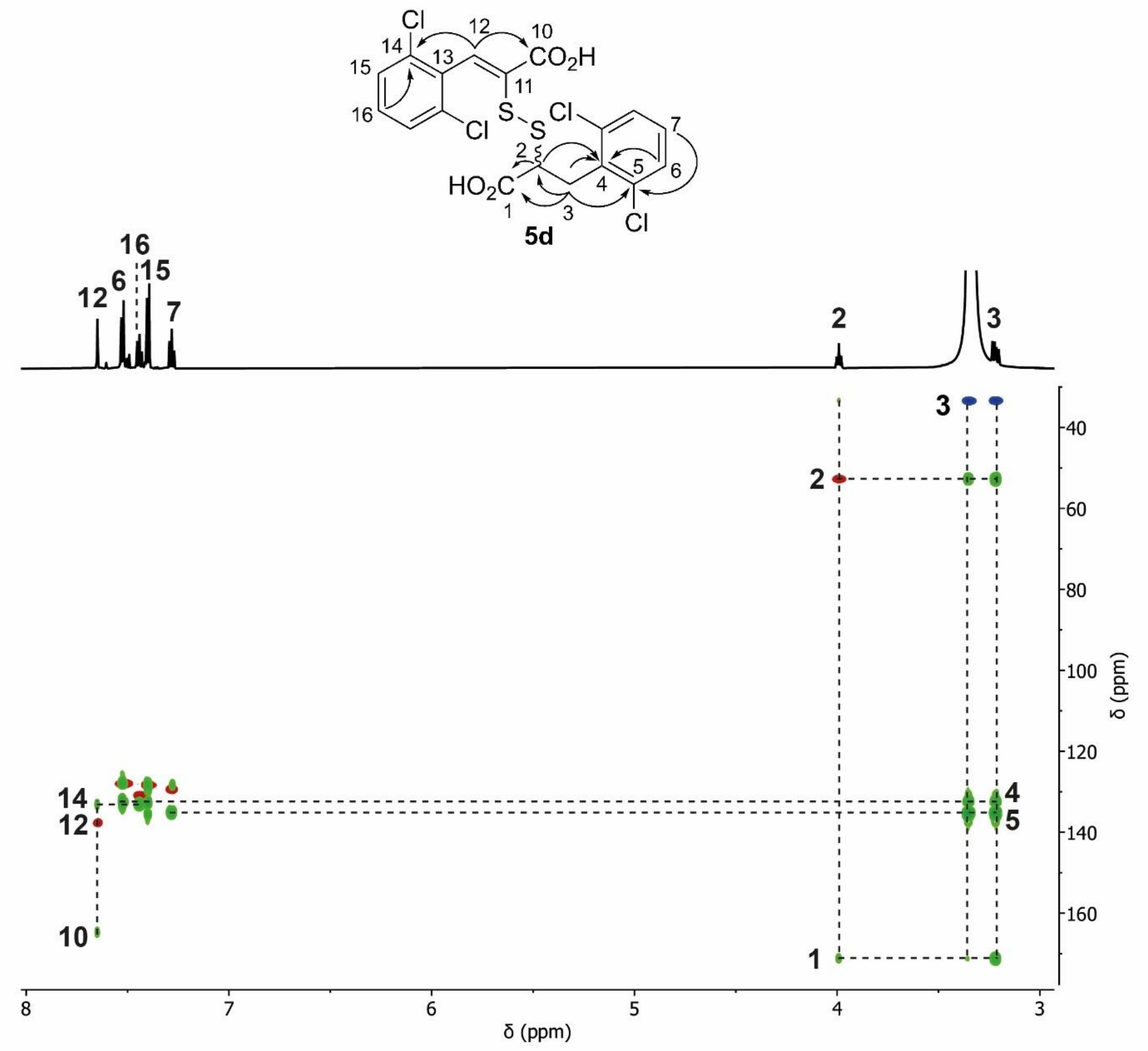

Figure S21: ^{1}H-^{13}C HSQC and HMBC NMR (700, 176 MHz, 298 K) characterisation of **5d**, the major product formed when **5b** (5 mM) reacts in DMSO-d$_6$ for 11 hours at room temperature. Cross peaks of the phase sensitive HSQC are in blue and red. Green peaks represent HMBC through bond correlations. Dashed lines represent through bond HMBC couplings, which are labelled by arrows in the structure. The arrows represent couplings and correspond to the dashed lines which highlight couplings used to assign **5d**.

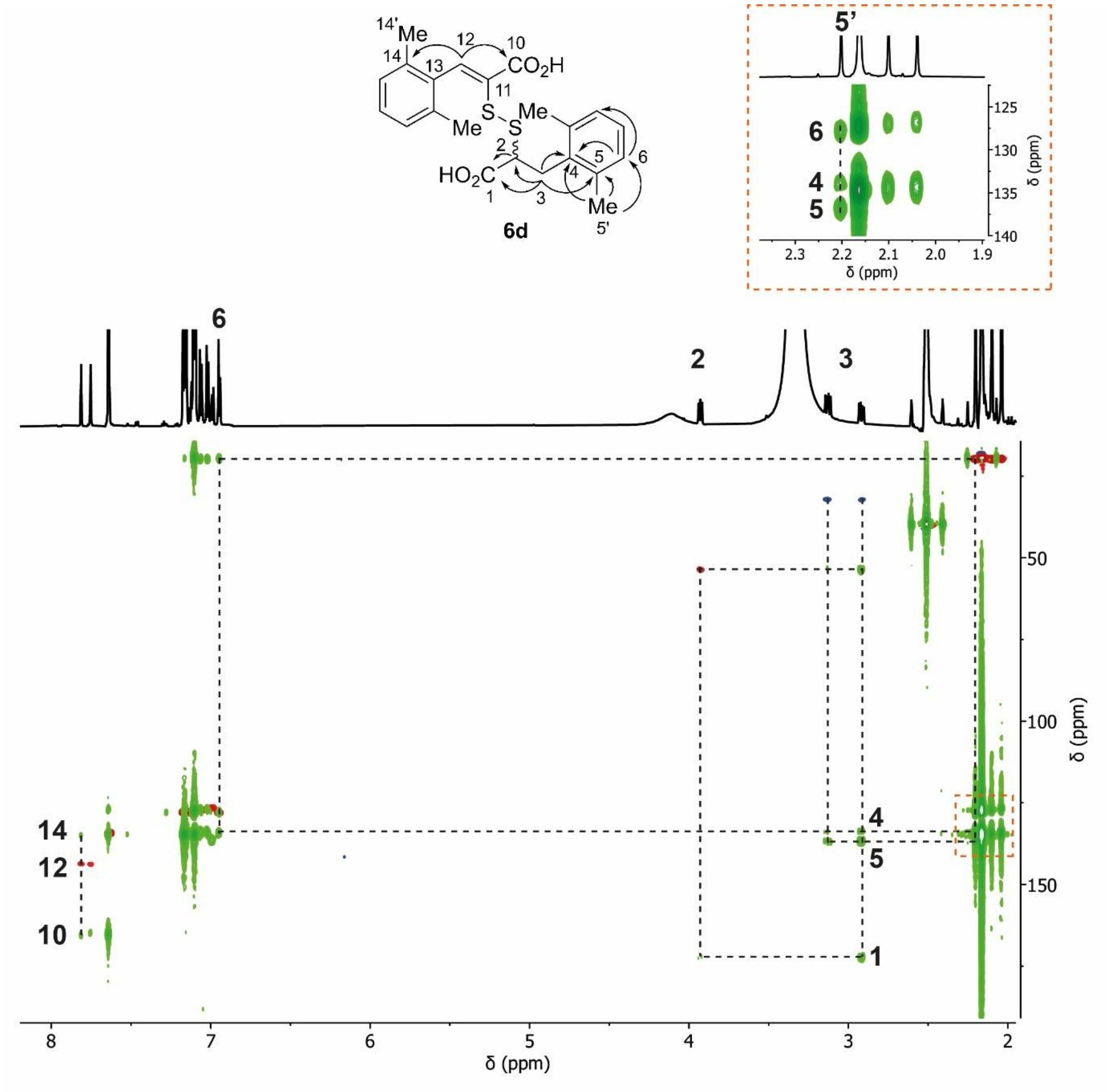

Figure S22: ^{1}H-^{13}C HSQC and HMBC NMR (700, 176 MHz, 298 K) characterisation of **6d**, the major product formed when **6b** (5 mM) reacts in DMSO-d_6 for 24 hours at room temperature. Cross peaks of the phase sensitive HSQC are in blue and red. Green peaks represent HMBC through bond correlations. The arrows represent couplings and correspond to the dashed lines which highlight couplings used to assign **6d**.

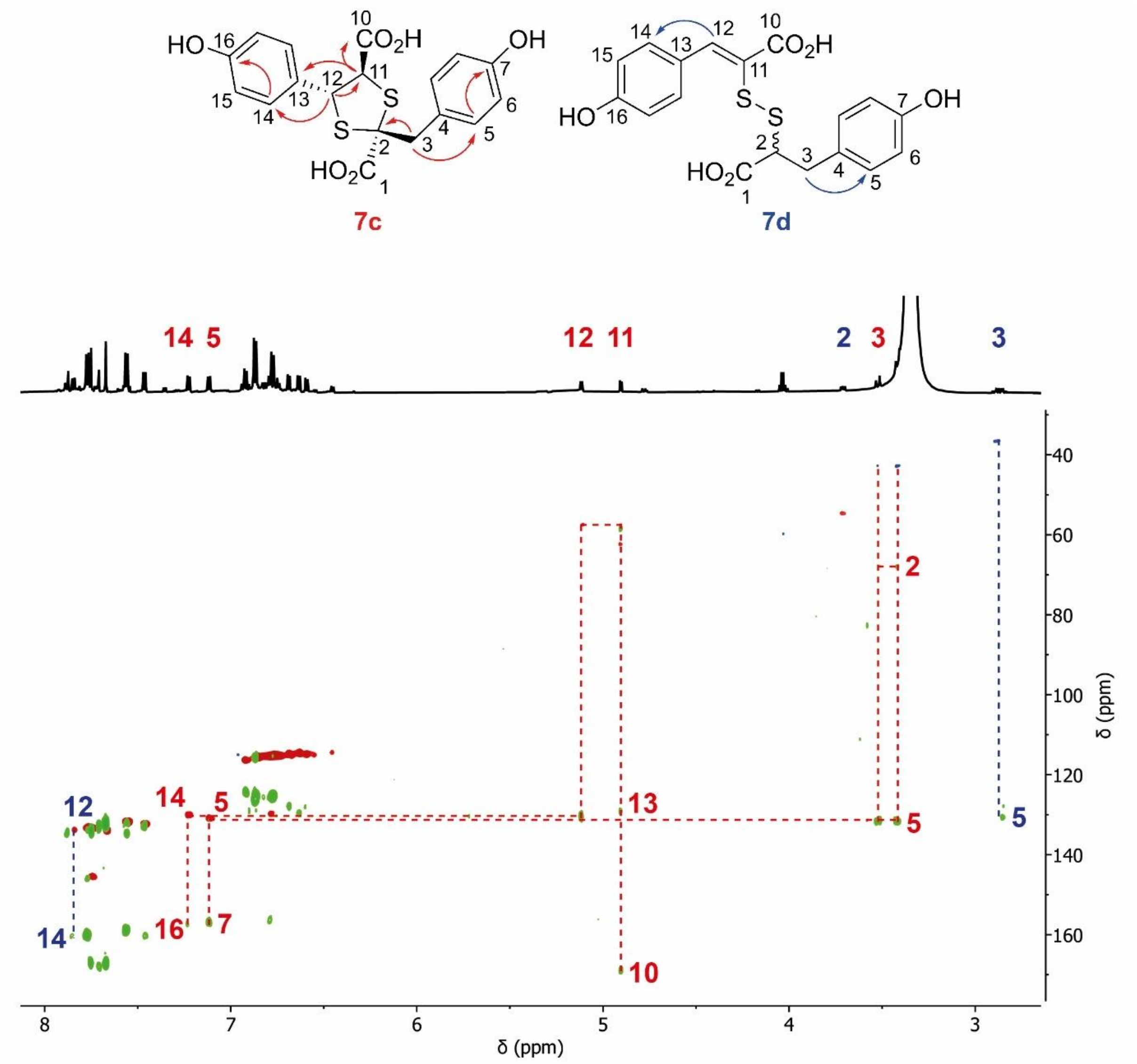

Figure S23: ^{1}H-^{13}C HSQC and HMBC NMR (700, 176 MHz, 298 K) characterisation of **7c** and **7d**, the major products formed when **7b** (5 mM) reacts in DMSO-d_6 for 24 hours at room temperature. Cross peaks of the phase sensitive HSQC are in blue and red. Green peaks represent HMBC through bond correlations. The arrows represent couplings and correspond to the dashed lines which highlight couplings used to assign **7c** and **7d**.

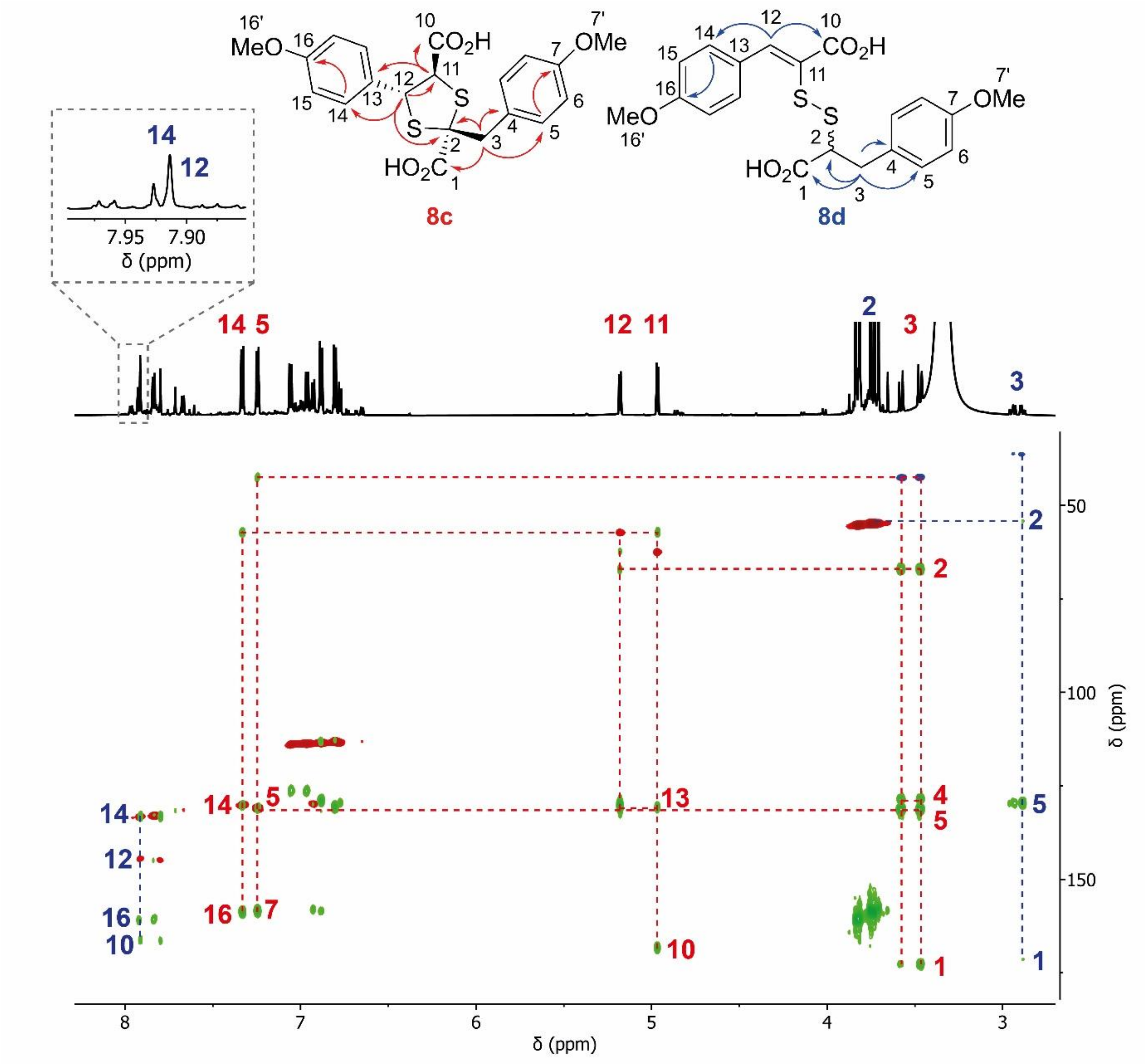

Figure S24: ^{1}H-^{13}C HSQC and HMBC NMR (700, 176 MHz, 298 K) characterisation of **8c** and **8d** the major products formed when **8b** (5 mM) reacts in DMSO-d_6 for 24 hours at room temperature. Cross peaks of the phase sensitive HSQC are in blue and red. Green peaks represent HMBC through bond correlations. The arrows represent couplings and correspond to the colour matched dashed lines which highlight couplings used to assign **8c** and **8d**.

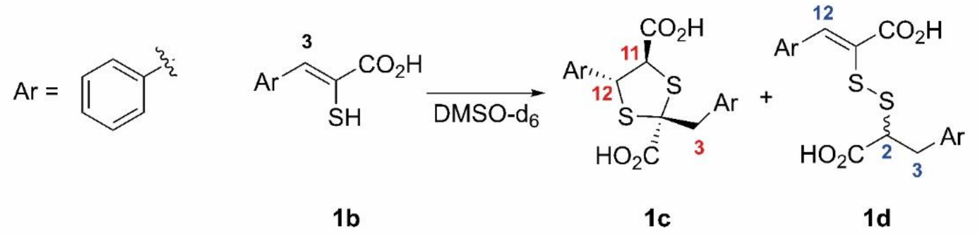

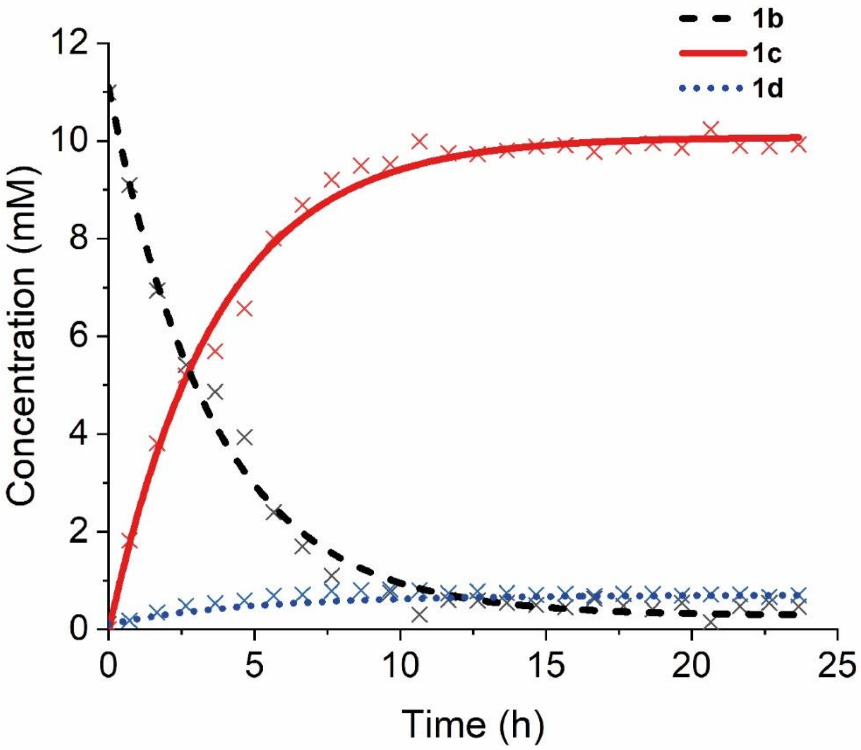

Figure S25: Reaction of **1b** (11 mM) in DMSO-d$_6$ at room temperature monitored over time, using ^{1}H NMR (700 MHz, 298 K). The plot is derived from relative integrals of non-overlapping resonances for **1b** (H-3), **1c** (H-12) and **1d** (H_2-3). Data were fitted using non-linear curve fits using OriginPro (2021b 9.8.5.212) with R^2 being 0.990 (enethiol, **1b**), 0.991 (1,3-dithiolane, **1c**), and 0.623 (mixed disulfide, **1d**).

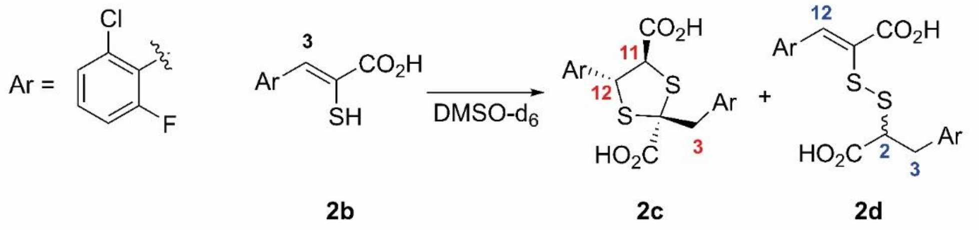

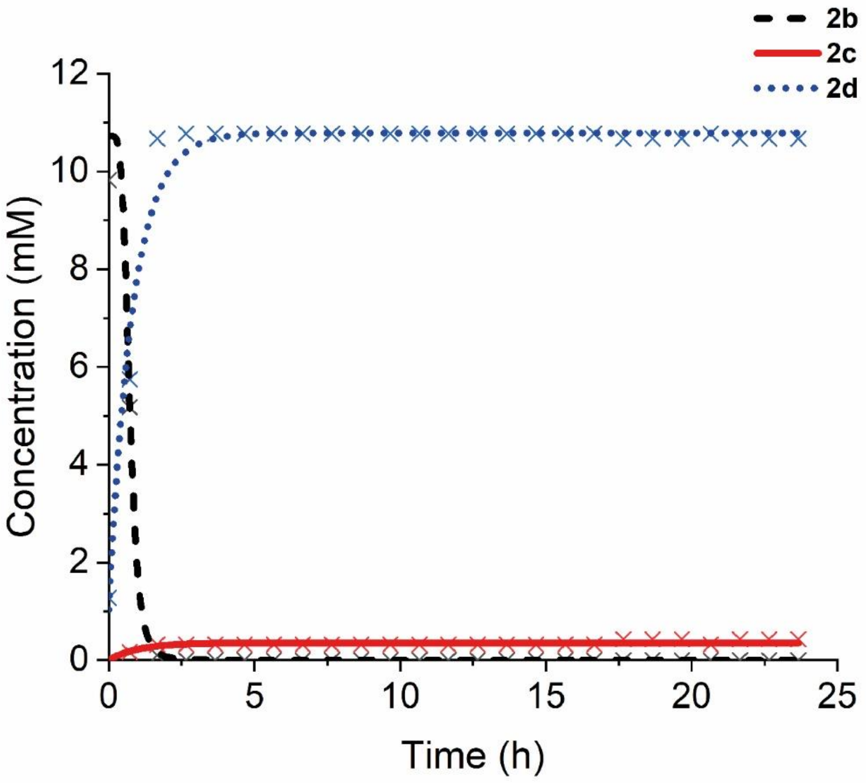

Figure S26: Reaction of **2b** (11 mM) in DMSO-d_6 at room temperature monitored over time, using ^{1}H NMR (700 MHz, 298 K). The plot is derived from relative integrals of characteristic non-overlapping resonances for **2b** (H-3), **2c** (H-12) and **2d** (H_2-3). Data were fitted using non-linear curve fits using OriginPro (2021b 9.8.5.212) with R^2 being 0.990 (enethiol, **2b**), 0.756 (1,3-dithiolane, **2c**), and 0.975 (mixed disulfide, **2d**).

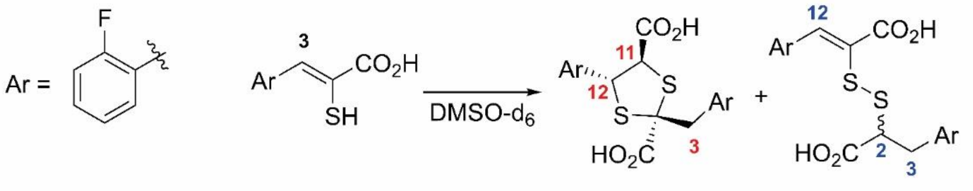

3b 3c 3d

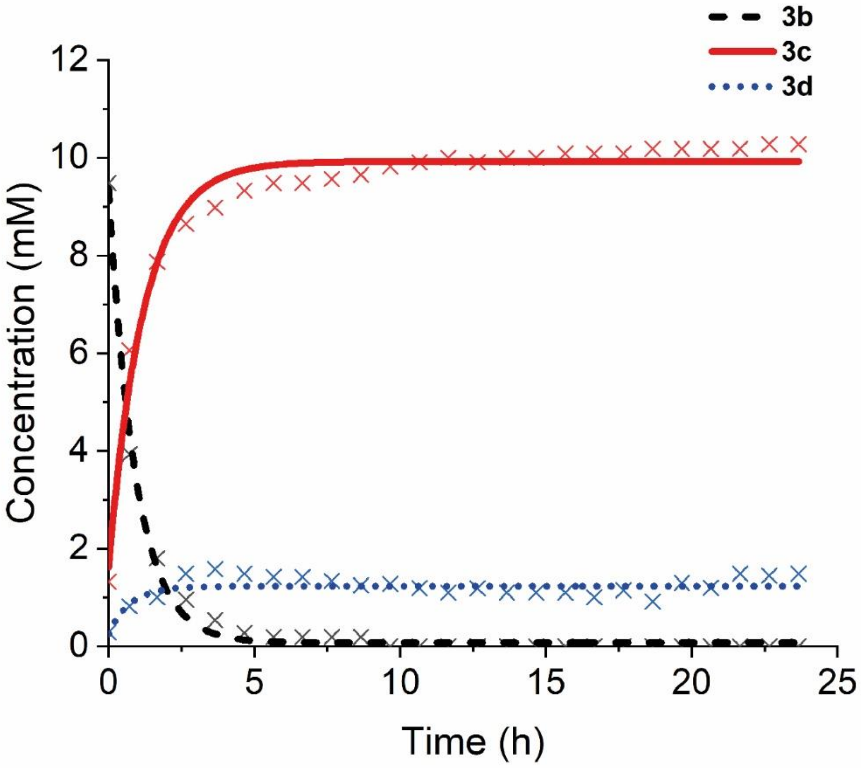

Figure S27: Reaction of **3b** (11 mM) in DMSO-d_6 monitored over time at room temperature, using ^{1}H NMR (700 MHz, 298 K). The plot is derived from relative integrals of characteristic non-overlapping resonances representing the precursor **3b** (H-3) and products **3c** (H-12) and **3d** (H_2-3). Data were fitted using non-linear curve fits using OriginPro (2021b 9.8.5.212) with R^2 being 0.994 (enethiol, **3b**), 0.974 (1,3-dithiolane, **3c**), and 0.571 (mixed disulfide, **3d**).

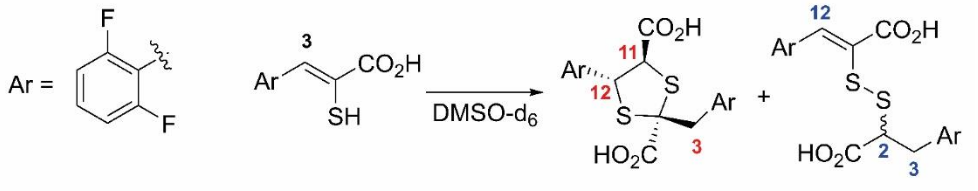

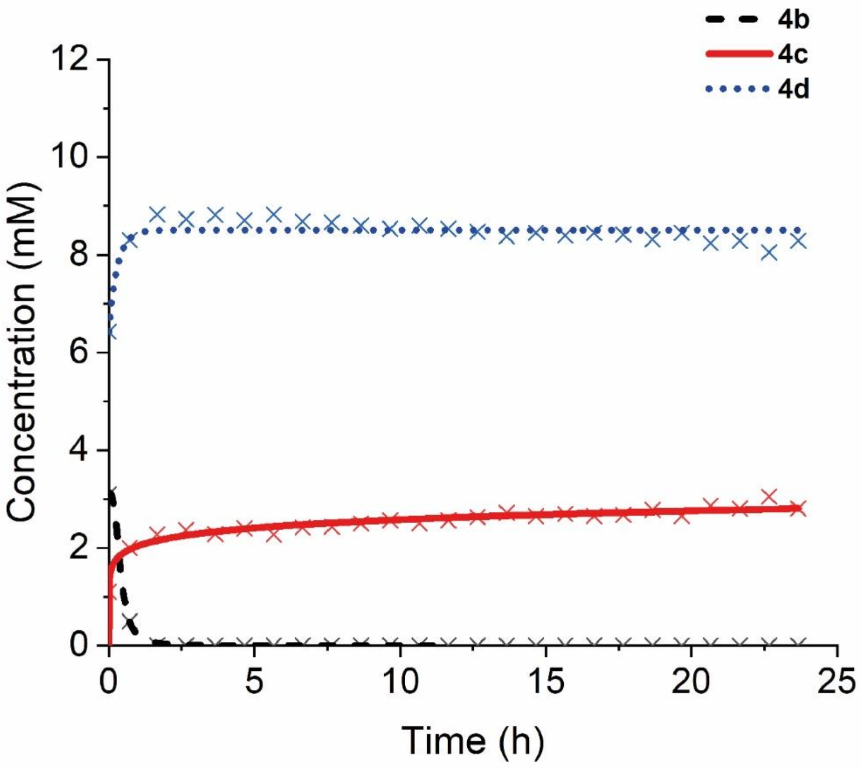

Figure S28: Reaction of **4b** (11 mM) in DMSO-d_6 at room temperature monitored over time, using ^{1}H NMR (700 MHz, 298 K). The plot is derived from relative integrals of characteristic non-overlapping resonances for **4b** (H-3), **4c** (H-12) and **4d** (H_2-3). Data were fitted using non-linear curve fits using OriginPro (2021b 9.8.5.212) with R^2 being 0.999 (enethiol **4b**), 0.911 (1,3-dithiolane, **4c**), and 0.820 (mixed disulfide, **4d**).

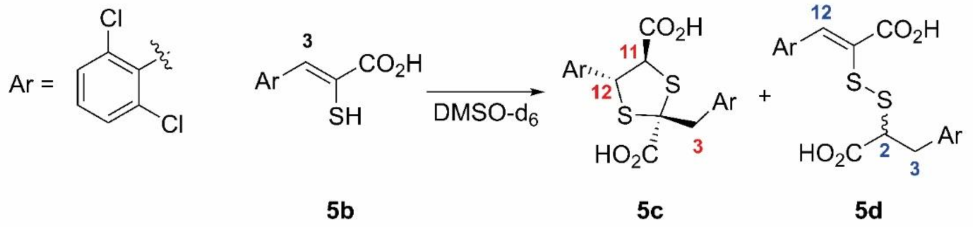

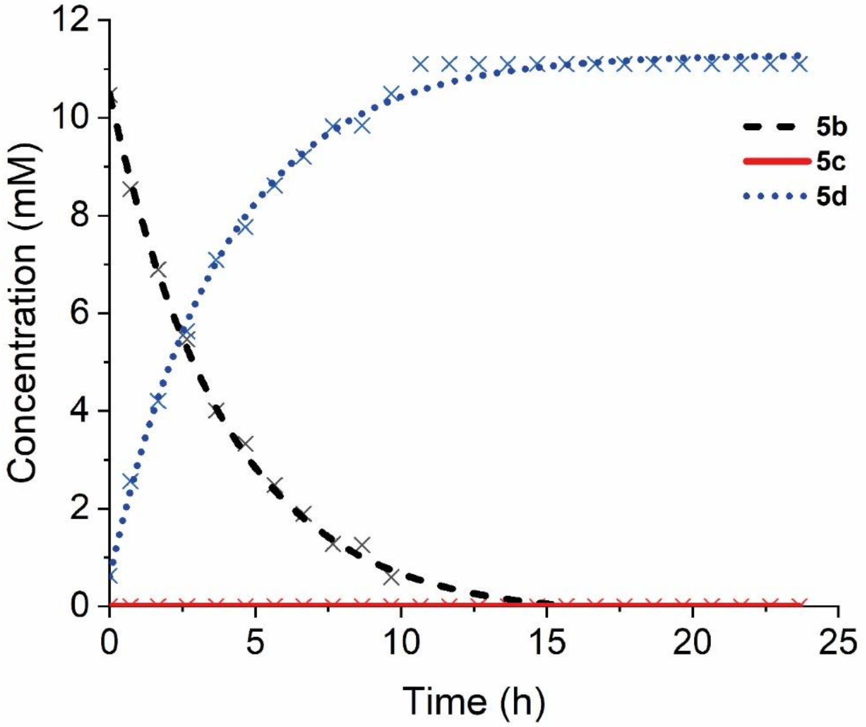

Figure S29: Reaction of **5b** (11 mM) in DMSO-d$_6$ at room temperature monitored over time, using ^{1}H NMR (700 MHz, 298 K). The plot is derived from relative integrals of characteristic non-overlapping resonances representing the precursor **5b** (H-3) and products **5c** (H-12) and **5d** (H_2-3). No evidence for formation of **5c** was observed. The data were fitted using linear and non-linear curve fits using OriginPro (2021b 9.8.5.212) with R^2 being 0.995 (enethiol, **5b**), 1.00 (1,3-dithiolane, **5c**), and 0.996 (mixed disulfide, **5d**).

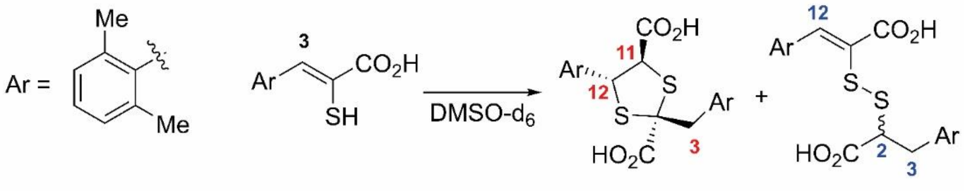

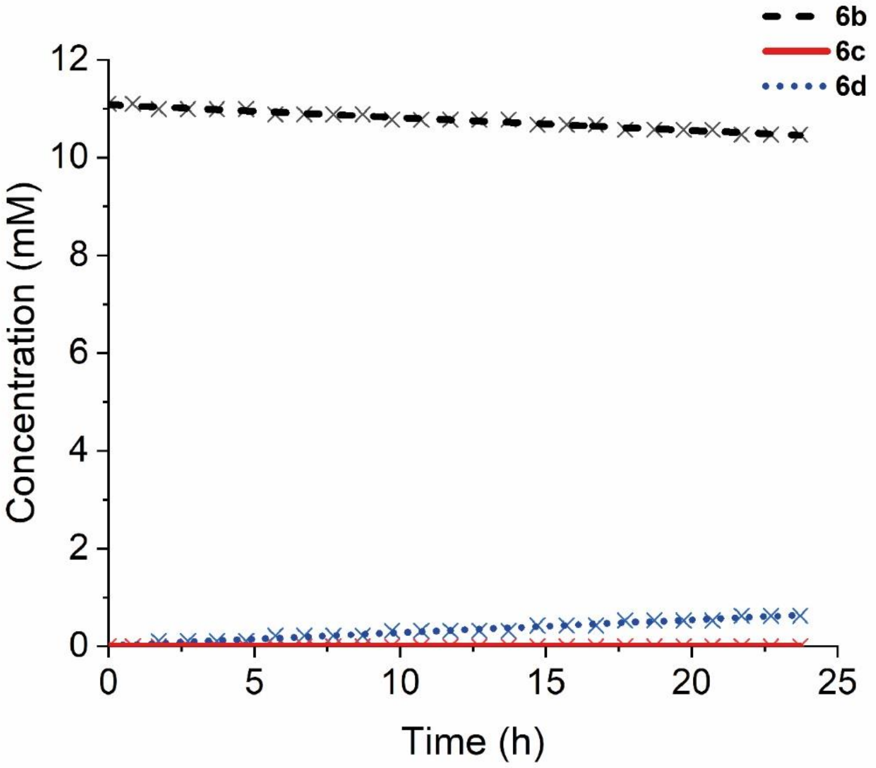

Figure S30: Reaction of **6b** (11 mM) in DMSO-d$_6$ at room temperature monitored over time, using ^{1}H NMR (700 MHz, 298 K). The plot is derived from relative integrals of characteristic non-overlapping resonances representing the precursor **6b** (H-3) and products **6c** (H-12) and **6d** (H_2-3). Note, no evidence for formation of **6c** was observed. The data were fitted using linear fits using OriginPro (2021b 9.8.5.212) with R^2 being 0.975 (enethiol, **6b**), 1.00 (1,3-dithiolane, **6c**), and 0.974 (mixed disulfide, **6d**).

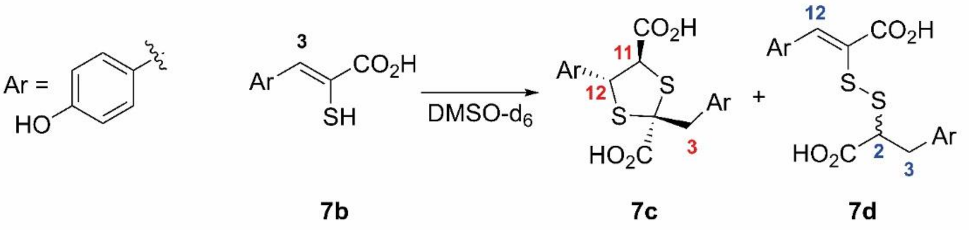

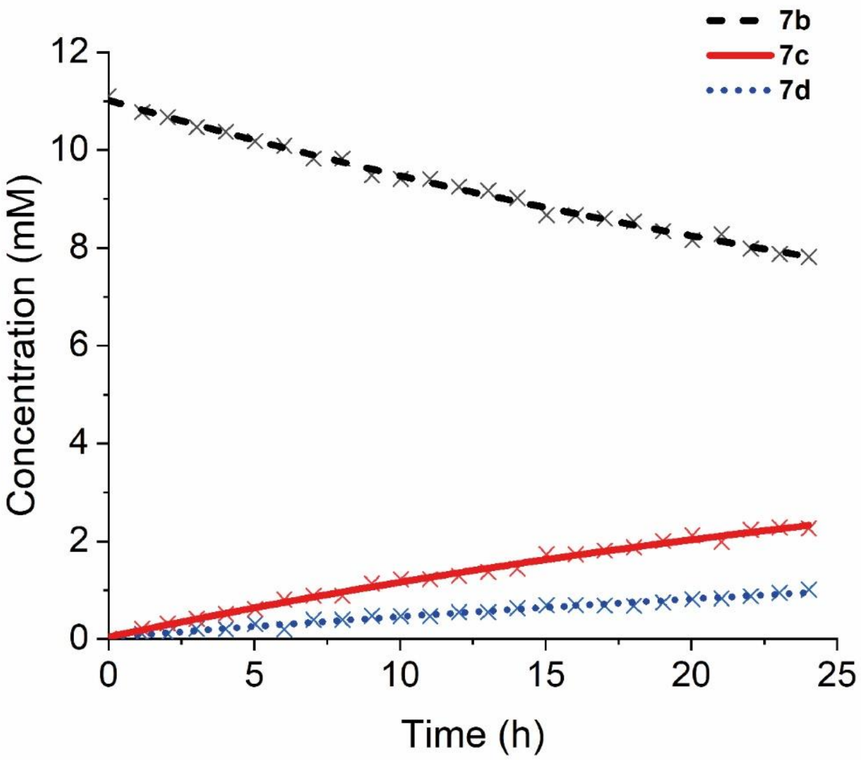

Figure S31: Reaction of **7b** (11 mM) in DMSO-d_6 at room temperature monitored over time, using ^{1}H NMR (700 MHz). The plot is derived from relative integrals of characteristic non-overlapping resonances for **7b** (H-3), **7c** (H-12) and **7d** (H_2-3). Data were fitted using non-linear curve fits using OriginPro (2021b 9.8.5.212) with R^2 being 0.994 (enethiol, **7b**), 0.993 (1,3-dithiolane, **7c**), and 0.980 (mixed disulfide, **7d**).

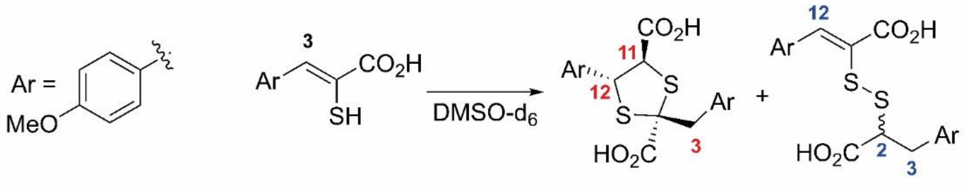

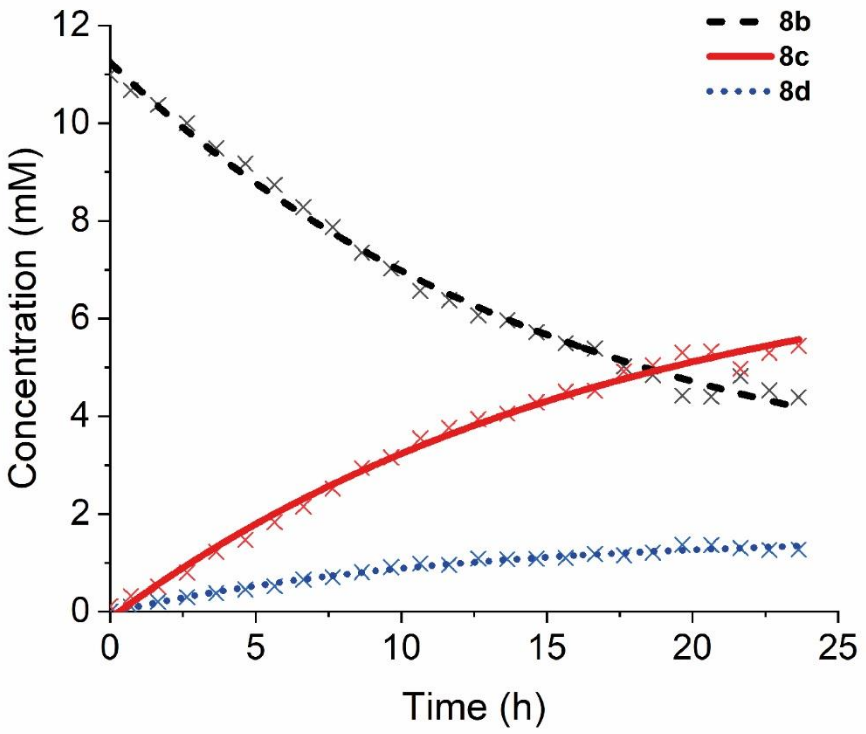

Figure S32: Reaction of **8b** (11 mM) in DMSO-d$_6$ at room temperature monitored over time, using ^{1}H NMR (700 MHz). The plot is derived from relative integrals of characteristic non-overlapping resonances representing the precursor **8b** (H-3) and products **8c** (H-12) and **8d** (H_2-3). Data were fitted using non-linear curve fits using OriginPro (2021b 9.8.5.212) with R^2 being 0.993 (enethiol, **8b**), 0.992 (1,3-dithiolane, **8c**), and 0.988 (mixed disulfide, **8d**).

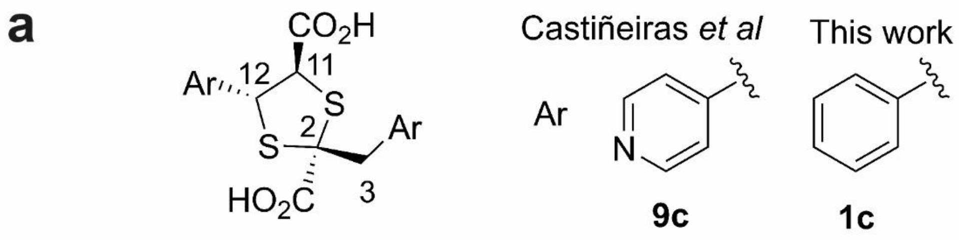

C (#)	δ (ppm)	
	Castiñeiras *et al* **9c**	This work **1c**
2	146.5	67.5
3	77.2	43.8
11	129.4	63.0
12	130.9	58.2

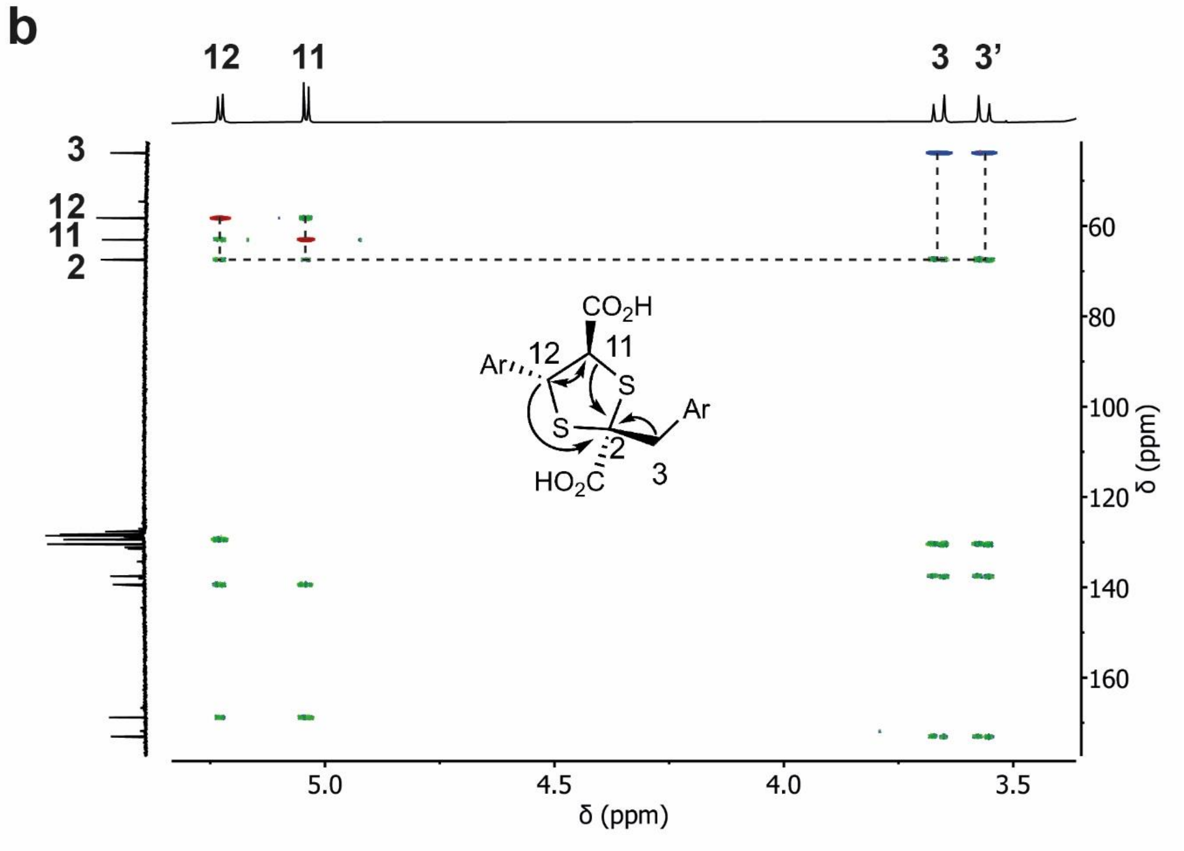

Figure S33: Comparison of the 1,3-dithiolane ring δ_C values reported by Castiñeiras *et. al.* for **9c** and those for **1c** assigned in this study.[1] a) Structure of 1,3-thiolane ring and comparison of δ_C (ppm) values. b) Overlay of HSQC (blue and red cross peaks) and HMBC (green cross peaks); couplings establishing the 1,3-thiolane ring structure are highlighted. Note that **1c** is used as an example for chemical shifts of the 1,3-dithiolane ring; similar chemical shifts are found of the other 1,3-dithiolane rings reported in this study.

General Experimental Conditions

The enethiols (**1-8b**) were prepared using reported methods and conditions.[2,3] Starting materials and products formed were monitored by NMR and characterized by 1D and 2D NMR techniques. Samples were recorded at 298 K, unless stated otherwise, in a 5 mm tube, using a Bruker AVII 500 equipped with a TXI H/F/C probe, Bruker AVIII HD 500 equipped with a BBF probe, Bruker AVIII HD 600 equipped with a BB-F/H N_2 CryoProbe or a Bruker AVIII HD 700 equipped with a TCI H/N/C He CryoProbe, at their respective resonances. Chemical shifts (δ) are reported in parts per million (ppm) referenced to the solvent resonance. Coupling constants (*J*) are reported to the nearest 0.5 Hz. Multiplicities are reported as singlet (s), doublet (d), triplet (t), multiplet (m). δ_C values are derived from 2D ^1H-^{13}C heteronuclear single quantum coherence spectroscopy (HSQC) and ^1H-^{13}C heteronuclear multiple bond correlation (HMBC) experiments. When specified, liquid chromatography mass spectrometry (LC-MS) was carried out using a Waters LCT Premier bench-top orthogonal acceleration time-of-flight (TOF) LC-MS system, equipped with a Acquite PDA detector. Reactions under anaerobic conditions (<2 ppm O_2) were performed in an anaerobic chamber (Belle technology, UK), under a N_2 atmosphere.

The standard reaction conditions for monitoring enethiol reactions were as follows. The solid enethiol was dissolved in DMSO-d_6 and diluted to give a solution (450 μL, 5 or 11 mM) of the desired concentration. The solution was transferred into an NMR tube (5 mm) and monitored over time. Reactions of enethiols in different solvents were studied in an analogous manner using the appropriate deuterated solvent, that is CD_3OD, 1,4-dioxane-d_8, DMF-d_7, THF-d_8, or acetone-d_6. For reactions carried out under anaerobic conditions, the solid enethiol, DMSO-d_6 (1 mL), and a J Young valve NMR tubes (5 mm, Norell, USA) were transferred to an anaerobic chamber (<2 ppm O_2, Belle Technology, UK) and left to equilibrate for 24 hours, prior to use. The solid enethiol was then dissolved in DMSO-d_6 and diluted appropriately (450 μL, 5 mM) before being transferred into the J Young valve NMR tube and monitored by ^1H NMR over time.

NMR Characterisation of products

(2*R*,4*R*,5*R*)-2-Benzyl-5-phenyl-1,3-dithiolane-2,4-dicarboxylic acid (1c)

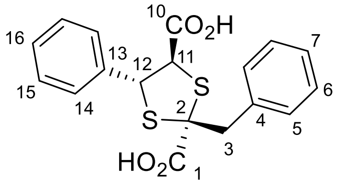

Reaction of **1b** (5.0 mM) in DMSO-d$_6$ resulted in the formation of **1c** as the major product (90%) observed after 10 hours.

^{1}H NMR (700 MHz, DMSO-d$_6$) δ: 7.42 (dd, *J* = 6.5, 3.0 Hz, 2H, H_{14}), 7.35 – 7.28 (m, 5H, H_5, and H_6, and H_7 or H_{16}), 7.28 – 7.21 (m, 3H, H_{15}, and H_7 or H_{16}), 5.22 (d, *J* = 6.5 Hz, 1H, H_{12}), 5.03 (d, *J* = 6.5 Hz, 1H, H_{11}), 3.66 (d, *J* = 14.0 Hz, 1H, H_3), 3.56 (d, *J* = 14.0 Hz, 1H, $H_{3'}$).

^{13}C NMR (151 MHz, DMSO-d$_6$) δ: 173.1 (C_1), 168.8 (C_{10}), 139.4 (C_{13}), 137.5 (C_4), 130.4 (C_5), 129.4 (C_{14}), 128.6 (C_6), 128.4 (C_{15}), 128.3 (C_{16}), 127.7 (C_7), 67.5 (C_2), 63.0 (C_{11}), 58.2 (C_{12}), 43.8 (C_3).

LC-MS (ESI$^+$, m/z): 361 ([M+H]$^+$, 100%).

(*Z*)-2-((1-Carboxy-2-phenylethyl)disulfaneyl)-3-phenylacrylic acid (1d)

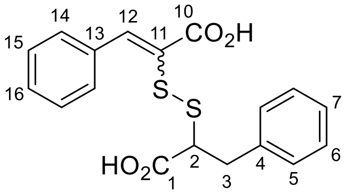

Reaction of **1b** (5.0 mM) resulted in the formation of **1d** as a minor product (6%) observed after 10 hours. The low abundance of **1c** precluded full assignment and characterisation.

Partial ^{1}H NMR (700 MHz, DMSO-d$_6$) δ: 7.93 (s, 1H, H_{12}), 7.83 (m, 4H, H_5 and H_{14}), 7.50 – 7.44 (m, 2H, H_7 and H_{16}), 7.31 – 7.28 (m, 2H, H_{16} and H_7), 3.80 (t, *J* = 8.0 Hz, 1H, H_2).

The relatively low abundance of **1d** precluded its detailed assignment / characterisation.

(2*R*,4*R*,5*R*)-5-Phenyl-2-(phenylmethyl-d)-1,3-dithiolane-2,4-dicarboxylic-4-d acid (1c-DD)

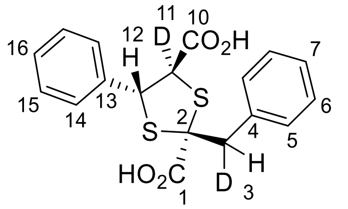

Reaction of **1a** (5.0 mM) in DMSO-d$_6$ (500 μL) supplemented with D_2O (20 μL), resulted in the formation of **1c-DD** as the major product (>75%) observed after 18 hours.

^{1}H NMR (600 MHz, DMSO-d$_6$) δ: 7.44 – 7.39 (m, 2H, H_{10}), 7.35 – 7.27 (m, 5H, H_5 and H_6, and H_7 or H_{16}), 7.27 – 7.22 (m, 3H, H_{15}, and H_7 or H_{16}), 5.22 (s, 1H, H_{12}), 3.64 (s, 1H, H_3), 3.54 (s, 1H, $H_{3'}$).

The relatively low abundance of **1c-DD** precluded its detailed assignment / characterisation.

(*Z*)-2-((1-Carboxy-2-(2-chloro-6-fluorophenyl)ethyl)disulfaneyl)-3-(2-chloro-6-fluorophenyl)acrylic acid (2d)

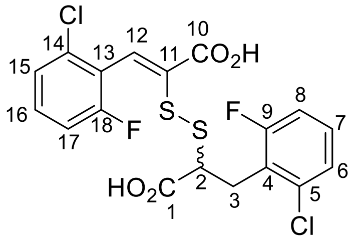

Reaction of **2b** (5.0 mM) in DMSO-d_6 resulted in the formation of **2d** as the major product (97%) observed after 2 hours.

^{1}H NMR (600 MHz, DMSO-d_6) δ: 7.61 (s, 1H, H_{12}), 7.53 – 7.48 (m, 1H, H_{16}), 7.41 (d, J = 8.0 Hz, 1H, H_{15}), 7.35 – 7.28 (m, 2H, H_7 and H_{17}), 7.25 (d, J = 8.0 Hz, 1H, H_6), 7.14 (t, J = 9.0 Hz, 1H, H_8), 3.83 (t, J = 8.0 Hz, 1H, H_2), 3.20 (dd, J = 14.0, 8.0 Hz, 1H, H_3), 3.04 (dd, J = 14.0, 8.0 Hz, 1H, $H_{3'}$).

^{13}C NMR (151 MHz, DMSO-d_6) δ: 171.5 (C_1), 165.2 (C_{10}), 161.5 (d, $^{1}J_{C\text{-}F}$ = 248.0 Hz, C_9), 159.3 (d, $^{1}J_{C\text{-}F}$ = 248.0 Hz, C_{18}), 135.0 (d, $^{3}J_{C\text{-}F}$ = 6.0 Hz, C_5), 134.3 (C_{12}), 133.8 (d, $^{3}J_{C\text{-}F}$ = 4.0 Hz, C_{14}) 132.2 (d, $^{3}J_{C\text{-}F}$ = 10.0 Hz, C_{16}), 130.1 (d, $^{3}J_{C\text{-}F}$ = 10.0 Hz, C_7), 125.9 (d, $^{4}J_{C\text{-}F}$ = 3.0 Hz, C_6), 125.8 (d, $^{4}J_{C\text{-}F}$ = 3.0 Hz, C_{15}), 125.8 (C_{11}), 123.4 (d, $^{2}J_{C\text{-}F}$ = 18.5 Hz, C_4), 122.1 (d, $^{2}J_{C\text{-}F}$ = 18.5 Hz, C_{13}), 115.1 (d, $^{2}J_{C\text{-}F}$ = 22.5 Hz, C_{17}), 114.8 (d, $^{2}J_{C\text{-}F}$ = 22.5 Hz, C_8), 53.3 (C_2), 29.4 (C_3).

^{19}F NMR (565 MHz, DMSO-d_6) δ: –107.5 (t, J = 7.0 Hz, F_{18} or F_9), –111.5 (t, J = 7.0 Hz, F_{18} or F_9).

LC-MS (ESI$^+$, m/z): (465 $[M+1]^+$, 100%).

(2*R*,4*R*,5*R*)-2-(2-Chloro-6-fluorobenzyl)-5-(2-chloro-6-fluorophenyl)-1,3-dithiolane-2,4-dicarboxylic acid (2c)

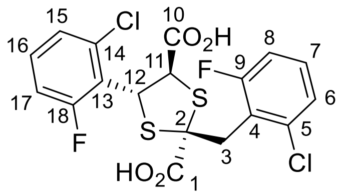

Reaction of **2b** (5.0 mM) in DMSO-d_6 resulted in the formation of **2c** as a minor product (~3%) observed over 2 hours.

Partial ^{1}H NMR (600 MHz, DMSO) δ: 7.49 – 7.45 (m, 2H, 2H), 7.37 (d, J = 7.5 Hz, 2H), 7.28 – 7.24 (m, 2H, H), 7.17 (d, J = 7.5 Hz, 2H), 5.63 (d, J = 6.5 Hz, H_{12}), 5.22 (d, J = 6.5 Hz, H_{11}), 3.71 – 3.66 (m, 1H, H_3), 3.61 – 3.57 (m, 1H, $H_{3'}$).

The relatively low abundance of **2c** precluded its detailed assignment / characterisation.

(2*R*,4*R*,5*R*)-2-(2-Fluorobenzyl)-5-(2-fluorophenyl)-1,3-dithiolane-2,4-dicarboxylic acid (3c)

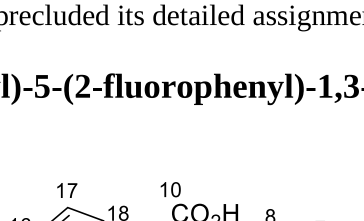

Reaction of **3b** (5.0 mM) in DMSO-d_6 (500 μL) resulted in the formation of **3c** as the major observed product (~93%) within an hour.

^{1}H NMR (700 MHz, DMSO-d_6) δ: 7.41 – 7.36 (m, 2H, H_5 and H_7), 7.26 – 7.13 (m, 4H, H_{18}, H_{16}, H_8, and H_7,), 7.08 (dd, J = 10.5, 8.5 Hz, 1H, H_{15}), 6.87 (t, J = 7.5 Hz, 1H, H_{17}), 5.42 (d, J = 6.5 Hz, 1H, H_{12}), 5.21 (d, J = 6.5 Hz, 1H, H_{11}), 3.60 (d, J = 14.6 Hz, 1H, H_3), 3.54 (d, J = 14.6 Hz, 1H, $H_{3'}$).

^{13}C NMR (151 MHz, DMSO-d$_6$) δ: 173.3 (C$_1$), 168.5 (C$_{10}$), 161.4 (d, $^1J_{C\text{-}F}$ = 244.5 Hz, C$_9$), 159.6 (d, $^1J_{C\text{-}F}$ = 246.0 Hz, C$_{14}$), 133.3 (d, $^3J_{C\text{-}F}$ = 3.5 Hz, C$_5$), 130.4 – 129.9 (m, 3C, C$_{18}$, C$_9$ and C$_7$) 127.0 (d, $^2J_{C\text{-}F}$ = 12.5 Hz, C$_{13}$), 124.5 (d, $^4J_{C\text{-}F}$ = 3.0 Hz, C$_{17}$), 124.3 (d, $^4J_{C\text{-}F}$ = 3.0 Hz, C$_6$), 123.7 (d, $^2J_{C\text{-}F}$ = 15.0 Hz, C$_4$), 115.6 (d, $^2J_{C\text{-}F}$ = 22.0 Hz, C$_8$), 115.1 (d, $^2J_{C\text{-}F}$ = 22.5 Hz, C$_{15}$), 66.9 (C$_2$), 62.8 (C$_{11}$), 49.0 (C$_{12}$), 34.6 (C$_3$).
The relatively low abundance of **2c** precluded its detailed assignment / characterisation.

(2*R*,4*R*,5*R*)-2-(2,6-Difluorobenzyl)-5-(2,6-difluorophenyl)-1,3-dithiolane-2,4-dicarboxylic acid (4c)

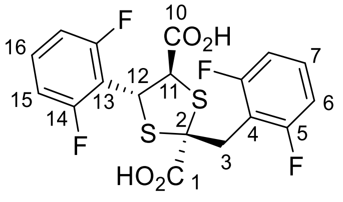

Reaction of **4b** (5.0 mM) in DMSO-d$_6$ (500 µL) resulted in the formation of **4c** as the minor product (~25%) observed within an hour.
^{1}H NMR (700 MHz, DMSO-d$_6$) δ: 7.57 – 7.52 (m, 1H, H$_{16}$), 7.35 – 7.30 (m, 1H, H$_7$), 7.19 (t, *J* = 8.0 Hz, 2H, H$_{15}$), 7.01 (t, *J* = 7.5 Hz, 2H, H$_6$), 5.43 (d, *J* = 7.5 Hz, H$_{12}$), 5.15 (d, *J* = 7.5 Hz, H$_{11}$), 3.56 (d, *J* = 15.0 Hz, H$_3$), 3.50 (d, *J* = 15.0 Hz, H$_{3'}$).
^{13}C NMR (176 MHz, DMSO-d$_6$) δ*: 172.6 (C$_1$) 168.5 (C$_{10}$),160.9 (C$_5$), 160.0 (C$_{14}$), 131.8 (C$_{16}$), 129.4 (C$_7$), 114.1 (C$_{13}$), 111.9 (C$_4$), 111.6 (C$_{15}$), 111.2 (C$_6$), 66.6 (C$_2$), 59.2 (C$_{11}$), 47.4 (C$_{12}$), 27.7 (C$_3$).
LC-MS (ESI$^-$, m/z): 431 ([M–H]$^-$, 100%).

(*Z*)-2-((1-Carboxy-2-(2,6-difluorophenyl)ethyl)disulfaneyl)-3-(2,6-difluorophenyl)acrylic acid (4d)

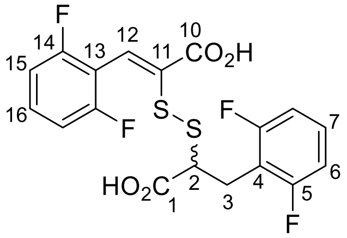

Reaction of **4b** (5.0 mM) in DMSO-d$_6$ (500 µL) resulted in the formation of **4d** as the major product (~75%) observed within an hour.
^{1}H NMR (700 MHz, DMSO-d$_6$) δ: 7.60 (s, 1H, H$_4$), 7.60 (s, 1H, H$_{12}$), 3.76 (t, *J* = 8.0 Hz, 1H, H$_2$), 3.08 (dd, *J* = 14.0 Hz, 1H, H$_3$), 2.93 (dd, *J* = 14.0 Hz, 1H, H$_{3'}$).
^{13}C NMR (176 MHz, DMSO-d$_6$) δ*: 171.1 (C$_1$), 164.8 (C$_{10}$), 160.8 (C$_5$), 129.4 (C$_7$), 158.6 (C$_{14}$), 131.8 (C$_{16}$), 130.4 (C$_{12}$), 114.3 (C$_{13}$), 112.6 (C$_4$), 111.6 (C$_{15}$), 111.2 (C$_6$), 52.7 (C$_2$), 24.6 (C$_3$).
LC-MS (ESI$^-$, m/z): 431 ([M–H]$^-$, 100%).
*Note that due to overlapping signals and the limited resolution available through 2D NMR data $J_{C\text{-}F}$ assignments were not made.

(*Z*)-2-((1-Carboxy-2-(2,6-dichlorophenyl)ethyl)disulfaneyl)-3-(2,6-dichlorophenyl)acrylic acid (5d)

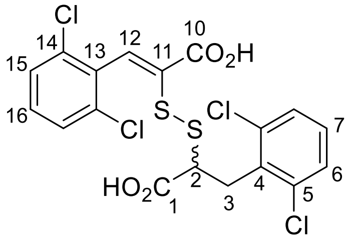

Reaction of **5b** (5.0 mM) in DMSO-d$_6$ (500 µL) resulted in formation of **5d** as the major product (complete conversion of **5b**) observed after 11 hours.
^{1}H NMR (700 MHz, DMSO-d$_6$) δ: 7.65 (s, 1H, H$_{12}$), 7.53 (d, *J* = 8.0 Hz, 2H, H$_{15}$), 7.44 (t, *J* = 8.0 Hz, 1H, H$_{16}$), 7.40 (d, *J* = 8.0 Hz, 2H, H$_6$), 7.28 (t, *J* = 8.0 Hz, 1H, H$_7$), 3.99 (t, *J* = 8.0 Hz, 1H, H$_2$), 3.35 (dd, *J* = 14.0, 8.0 Hz, 1H, H$_3$), 3.22 (dd, *J* = 14.0, 8.0 Hz, 1H, H$_{3'}$)
^{13}C NMR (176 MHz, DMSO-d$_6$) δ: 171.2 (C$_1$), 164.7 (C$_{10}$), 137.7 (C$_{12}$), 135.2 (C$_5$),133.2 (C$_{14}$), 132.8 (C$_4$), 132.3 (C$_{13}$), 130.9 (C$_{16}$), 129.4 (C$_7$), 128.3 (C$_6$), 128.1 (C$_{15}$), 52.7 (C$_2$), 33.3 (C$_3$).
LC-MS (ESI$^+$, m/z): 497 [M+1]$^+$.

(*Z*)-2-((1-Carboxy-2-(2,6-dimethylphenyl)ethyl)disulfaneyl)-3-(2,6-dimethylphenyl)acrylic acid (6d)

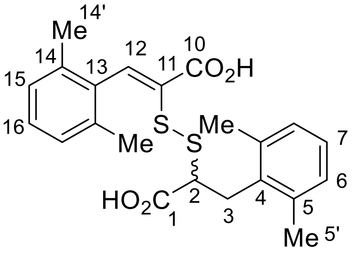

Reaction of **6b** (5.0 mM) resulted in the formation of **6d** as the major product, with low conversion (~6%) observed after 24 hours.
^{1}H NMR (600 MHz, DMSO-d$_6$) δ: 7.81 (s, 1H, H$_{12}$), 7.18 – 7.09 (m, 1H, H$_{16}$), 7.05 (d, *J* = 7.5 Hz, 2H, H$_{15}$) 7.01 – 6.97 (m, 1H, H$_7$), 6.94 (d, *J* = 7.0 Hz, 2H, H$_6$), 3.91 (dd, *J* = 9.5, 6.5 Hz, 1H, H$_2$), 3.12 (dd, *J* = 14.0, 9.5 Hz, 1H, H$_3$), 2.91 (dd, *J* = 14.0, 6.5 Hz, 1H, H$_{3'}$), 2.19 (s, 6H, H$_{5'}$), 2.03 (s, 6H, H$_{14'}$).
^{13}C NMR (151 MHz, DMSO-d$_6$) δ: 172.8 (C$_1$), 165.9 (C$_{10}$), 144.2 (C$_{12}$), 137.1 (C$_5$), 135.2 (C$_4$), 135.1 (C$_{14}$), 134.5 (C$_{13}$), 128.6 (C$_6$), 128.3 (C$_{16}$), 127.7 (C$_{15}$), 127.0 (C$_7$), 54.2 (C$_2$), 32.7 (C$_3$), 20.3 (C$_{5'}$ and C$_{14'}$).
LC-MS (ESI$^-$, m/z): 207 ([M–2]$^-$, 100%), 415 ([M–1]$^+$, 5%).

(2*R*,4*R*,5*R*)-2-(4-Hydroxybenzyl)-5-(4-hydroxyphenyl)-1,3-dithiolane-2,4-dicarboxylic acid (7c)

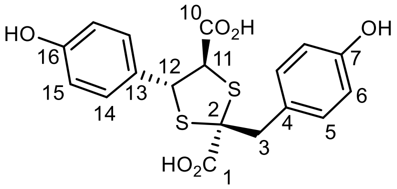

Reaction of **7b** (5.0 mM) resulted in the formation of **7d** as the minor product, with low conversion (~20%) observed after 24 hours.
^{1}H NMR (700 MHz, DMSO-d$_6$) δ: 7.22 (d, *J* = 8.5, 2H, H$_{14}$), 7.11 (d, *J* = 8.5 Hz, 2H, H$_5$), 6.68 (d, *J* = 8.5 Hz, 2H, H$_6$) 6.63 (d, *J* = 8.5 Hz, 2H, H$_{15}$), 5.11 (d, *J* = 6.5 Hz, 1H, H$_{12}$), 4.90 (d, *J* = 6.5 Hz, 1H, H$_{11}$), 3.53 – 3.49 (m, 1H, H$_3$), 3.43 – 3.39 (m, 1H, H$_{3'}$).

^{13}C NMR (176 MHz, DMSO-d$_6$) δ: 173.4 (C_{1}), 169.0 (C_{10}), 157.3 (C_{16}), 157.1 (C_{7}), 131.4 (C_{5}), 130.6 (C_{14}), 129.3 (C_{13}), 128.0 (C_{4}), 115.3 (C_{6}), 115.0 (C_{15}), 67.8 (C_{2}), 63.0 (C_{11}), 58.2 (C_{12}), 43.2 (C_{3}).
The low abundance of **7c** precluded its detailed assignment / characterisation.

(*Z*)-2-((1-Carboxy-2-(4-hydroxyphenyl)ethyl)disulfaneyl)-3-(4-hydroxyphenyl)acrylic acid (7d)

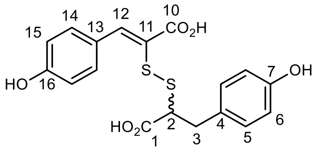

Reaction of **7b** (5.0 mM) resulted in the formation of **7d** as a minor product, with low conversion (~9%) over 24 hours.
^{1}H NMR (700 MHz, DMSO-d$_6$) δ: 7.87 (s, 1H, H_{12}), 3.74 – 3.66 (m, 1H, H_{2}), 2.93 – 2.82 (m, 2H, H_{3}).
^{13}C NMR (176 MHz, DMSO-d$_6$) δ: 167.5 (C_{10}), 145.2 (C_{12}), 134.4 (C_{14}), 54.6 (C_{2}), 36.4 (C_{3}).
The relatively low abundance of **7d** precluded its detailed assignment / characterisation.

(2*R*,4*R*,5*R*)-2-(4-Methoxybenzyl)-5-(4-methoxyphenyl)-1,3-dithiolane-2,4-dicarboxylic acid (8c)

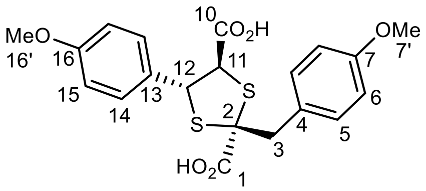

Reaction of **8b** (5.0 mM) resulted in the formation of **8c** as the major product, with incomplete conversion (~49%) observed after 24 hours.
^{1}H NMR (700 MHz, DMSO-d$_6$) δ: 7.33 (d, J = 8.5 Hz, 2H, H_{14}), 7.25 (d, J = 8.5 Hz, 2H, H_{5}), 6.90 – 6.88 (m, 2H, H_{6}), 6.82 – 6.77 (m, 2H, H_{15}), 5.18 (d, J = 6.0 Hz, 1H, H_{12}), 4.97 (d, J = 6.0 Hz, 1H, H_{11}), 3.75 (s, 3H, $H_{7'}$), 3.73 (s, 3H, $H_{16'}$).
^{13}C NMR (176 MHz, DMSO-d$_6$) δ: 172.6 (C_{1}), 168.3 (C_{10}), 158.7 (C_{16}), 158.4 (C_{7}), 130.1 (C_{14}), 131.0 (C_{5}), 130.7 (C_{13}), 129.0 (C_{4}), 113.4 (C_{6}), 113.1 (C_{15}) 67.1 (C_{2}) 62.6 (C_{11}), 57.3 (C_{12}), 54.9 ($C_{7'}$ and $C_{16'}$), 42.5 (C_{3}).
LC-MS (ESI$^-$, m/z): 419 ([M–H]$^-$, 100%).

(*Z*)-2-((1-Carboxy-2-(4-methoxyphenyl)ethyl)disulfaneyl)-3-(4-methoxyphenyl)acrylic acid (8d)

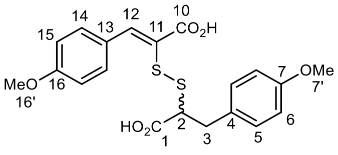

Reaction of **8b** (5.0 mM) resulted in the formation of **8d** as the minor product, with low conversion observed (~11%) observed after 24 hours.
^{1}H NMR (700 MHz, DMSO-d$_6$) δ: 7.84 (d, J = 9.0 Hz, 2H, H_{14}), 7.80 (s, 1H, H_{12}), 6.98 – 6.95 (m, 2H, H_{15}), 6.94 – 6.91 (m, 2H, H_{5}), 6.79 – 6.76 (m, 2H, H_{6}), 3.82 (s, 3H, $H_{16'}$), 3.80 – 3.74 (m, 1H, H_{2}), 3.71 (s, 3H, $H_{7'}$).
^{13}C NMR (176 MHz, DMSO-d$_6$) δ: 171.4 (C_{1}), 166.4 (C_{10}), 160.7 (C_{116}), 144.9 (C_{12}), 133.0 (C_{14}), 129.9 (C_{5}), 129.7 (C_{4}), 126.4 (C_{13}), 113.7 (C_{15}), 113.4 (C_{6}), 55.3 ($C_{16'}$), 54.8 ($C_{7'}$) 54.2 (C_{2}), 36.4 (C_{3}).
The relatively low abundance of **8d** precluded its detailed assignment / characterisation.

References

1. Castiñeiras, A., Gil, M. J. & Sevillano, P. Synthesis, spectroscopic characterization and X-ray crystal structure of 2-(4-methylpyridinium)-5-(4-pyridinium)-1,3-dithiolane-2,4-dicarboxylic acid dithiocyanate monohydrate. *J Mol Struct* **522**, 193–199 (2000).

2. Brem, J. *et al.* Rhodanine hydrolysis leads to potent thioenolate mediated metallo-β 2-lactamase inhibition. *Nat Chem* **6**, 1084–1090 (2014).

3. Zhang, D. *et al.* Structure activity relationship studies on rhodanines and derived enethiol inhibitors of metallo-β-lactamases. *Bioorg Med Chem* (2018) doi:10.1016/j.bmc.2018.02.043.
